# Domino Synthesis of 1,2,5-Trisubstituted 1*H*-Indole-3-carboxylic Esters Using a [3+2] Strategy

**DOI:** 10.3390/molecules30030444

**Published:** 2025-01-21

**Authors:** Siddhartha Maji, Kwabena Fobi, Ebenezer Ametsetor, Richard A. Bunce

**Affiliations:** Department of Chemistry, Oklahoma State University, Stillwater, OK 74078-3071, USA; smaji@okstate.edu (S.M.); kfobi@okstate.edu (K.F.); eametse@okstate.edu (E.A.)

**Keywords:** domino reactions, [3+2] anionic cyclization, imine addition, S_N_Ar ring closure, 1,2,5-trisubstituted 1*H*-indole-3-carboxylic esters, heterocycle synthesis

## Abstract

A new approach to 1,2,5-trisubstituted 1*H*-indole-3-carboxylic esters has been developed and studied. The method begins with the preparation of imines from aldehyde and primary amine derivatives. Treatment of these imines with the K_2_CO_3_-derived anion from methyl 2-(2-fluoro-5-nitrophenyl)acetate or methyl 2-(5-cyano-2-fluorophenyl)acetate in DMF initiates a [3+2] cyclization by addition of the anion to the imine followed by ring closure of the adduct nitrogen to the activated aromatic moiety via an S_N_Ar process. Twenty-one examples are reported. Temperatures required for the conversion range from 90 to 95 °C for the nitro-activated substrates to 125 to 130 °C for the cyano-activated precursors. Though efficient and atom economical, limitations arise from steric hindrance in the reacting partners. The initial indoline formed is not observed but instead undergoes spontaneous air oxidation to the give the aromatic heterocycle. Imines from nonaromatic aldehydes and amines are also possible, but these give slightly lower yields of 1*H*-indoles and only react with the nitro-activated substrates. The results are presented with a discussion of the mechanism and the factors important to the success of the reaction.

## 1. Introduction

Indoles comprise an important class of heterocycles that are ubiquitous in nature, and many express significant and wide-ranging biological activities. Early methods to assemble the basic ring system have been known for over 100 years [1]. Since that time, new approaches have been added, and myriad improvements have been made to increase the efficiency of the cyclizations as well as to functionalize the rings [2,3,4].

**Scheme 1:**
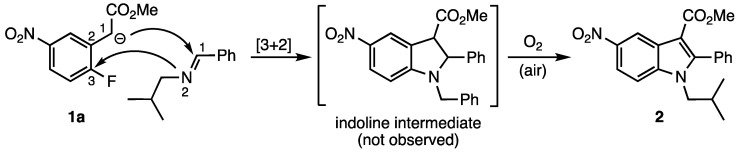
The [3+2] conversion of the anion of methyl 2-fluoro-5-nitrophenylacetate (**1a**) and *N*-isobutyl-1-phenylmethanimine to give methyl 1-isobutyl-2-phenyl-1*H*-indole-3-carboxylate (**2**).

Our previous syntheses of indoles introduced two domino strategies: the first involved a reduction-addition-elimination sequence, and the second an aza-Michael-S_N_Ar-aromatization process. The first required initial S_N_Ar addition of acetoacetic ester anions to 2-fluoronitrobenzene to give 2-(2-nitrophenyl)-substituted β-ketoesters. Subsequent reduction of the nitro group initiated a domino sequence involving addition of the aniline nitrogen to the conjugated enolic double bond and elimination of water to afford a 2-alkyl-1*H*-indole-3-carboxylic ester [5]. This ring closure was essentially a Leimgruber-Batcho synthesis modified to eliminate water and provide indole-3-carboxylic esters [6]. The second method began with aza-Michael addition of amines to activated methyl 2-(2-fluoroaryl)acrylate esters and concluded with S_N_Ar ring closure and aromatization to produce 1-alkyl-1*H*-indole-3-carboxylate esters [7]. The current paper discloses a new, one-pot, anionic [3+2] approach to methyl 1,2,5-trisubstituted-1*H*-indole-3-carboxylates from S_N_Ar-activated methyl (2-fluorophenyl)acetate derivatives by (1) addition of the ester anion to an imine C=N, (2) S_N_Ar cyclization of the adduct nitrogen to the activated aromatic moiety, and (3) air oxidation (Scheme 1). Precedent for the imine addition-S_N_Ar ring closure was first described by this laboratory in a previous synthesis of 4-oxo-1,2,3,4-tetrahydroquinoline-3-carboxylic esters [8].

The classical route to prepare 1*H*-indole-3-carboxylic esters is the Nenitzescu synthesis, which involves conjugate addition of a primary or secondary vinylogous amide to benzoquinone, followed by cyclocondensation [9]. To date, only one synthesis of 1*H*-indoles, developed in this laboratory, has utilized a S_N_Ar reaction to close the nitrogen-containing ring of the fused structure [7]. The present work uses this same ring-closure strategy, proceeds in one operation with no metals, and requires only base and heat in *N*,*N*-dimethylformamide (DMF) solvent. The advantage of this route derives from its efficiency, atom economy [10,11] and convenient chromatographic purification to give a product with no residual metal contaminants.

Transition metal catalysts based on copper, palladium, and rhodium have all been exploited to generate indole-3-carboxylate esters, though several of the procedures appeared to be quite complicated. Punniyamurthy advanced a ligand-free copper(I)-catalyzed synthesis of these targets from 2-iodoanilines and 1,3-dicarbonyl compounds in the presence of Cs_2_CO_3_ in aqueous DMSO at 100 °C [12]. Peng and co-workers produced similar structures via a Cu(I)/Johnphos-promoted C-to-N linking of an iodoarene with an enamine to give an *N*-aryl enamine, followed by intramolecular cross dehydrogenative coupling (CDC) of the enamine carbon with C2 of the aromatic ring in DMSO at 130 °C [13]. These researchers also summarized additional approaches to these heterocycles that used the CDC strategy with other copper and palladium catalysts [13]. Palladium complexes have also found use in the preparation of indole-3-carboxylic esters. Kim and Lee employed Ph_4_Pd and NaHMDS in DMSO at 120 °C to construct indoles using Blaise reaction intermediates derived from nitriles and ethyl 2-bromo-(2-bromophenyl)acetate [14]. The Varwani group assembled the target system from various methyl 3-(benzylamino)-2-(bromophenyl)-2-butenoate precursors in the presence of the RuPhos gen (III) Pd catalyst system and NaOMe in 1,4-dioxane at 100 °C [15]. The Zhu group described a [RhCp*Cl_2_]_2_-catalyzed route to indoles from *N*-nitrosoanilines and alkynes that used the N–N bond as an oxidant in a redox neutral process [16]. Using this same rhodium(III) catalyst with CsOAc in acetic acid-dichloroethane, Li and co-workers generated indoles from imidamides and diazoketoesters [17]. A final article from the Wu laboratory synthesized the indole ester via a catalytic *fac-*Ir(ppy)_3_-photosensitized intramolecular radical ring closure of ethyl 3-aryl-3-(phenylamino)acrylates under aerobic conditions in DMSO at 75 °C [18].

Metal-free protocols to prepare indole-3-carboxylate esters have also been reported. Doyle and Zhou produced C2-substituted derivatives using a BF_3_–Et_2_O-catalyzed intramolecular cyclization of methyl phenyldiazoacetates bearing C2 phenylimino groups [19]. Levesque and Fournier used this same Lewis acid with ethyl diazoacetate and 2-aminobenzaldehydes to generate indoles by a sequence that entailed addition to the aldehyde, a 1,2-aryl shift, and condensative ring closure [20]. Zhao and co-workers accessed the target esters via a phenyliodine bis(trifluoroacetate) (PIFA)-mediated intramolecular cyclization of ethyl 3-(arylamino)-2-phenyl-2-butenoate derivatives [21]. A later study by the Karchava group employed KO-*t*-Bu in DMF in an electron-catalyzed intramolecular cyclization of 3-amino-2-(2-bromophenyl)acrylates [22]. Finally, the Greaney team prepared indole-3-carboxylic esters in two steps by reaction of benzynes with tosylhydrazones and subsequent Fischer indole synthesis [23].

**Figure 1:**
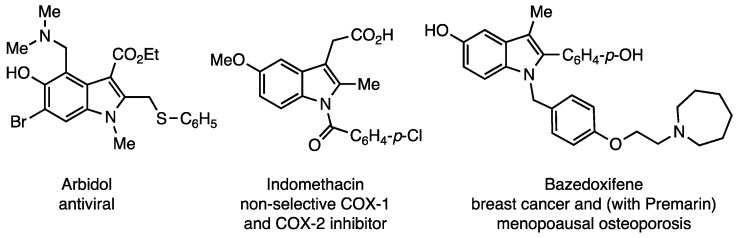
Medicinal agents incorporating highly substituted 1*H*-indoles.

Indoles are well represented in the pantheon of drug molecules, and this has been extensively reviewed [24,25,26]. Several derivatives which exhibit substitution patterns similar to those available by the current method are pictured in Figure 1 below. Arbidol is a broad-spectrum antiviral agent that has demonstrated potential against influenza A, B, and C, respiratory syncytial virus (RSV), herpes simplex, and hepatitis B and C, among others [27]. Indomethacin, a non-selective COX-1 and COX-2 inhibitor, displays potent NSAID activity by blocking the production of prostaglandins, which contribute to painful inflammation caused by arthritis and gout [28]. Finally, Bazedoxifene in combination with Premarin has been approved for the treatment of menopausal osteoporosis and is being further investigated as a possible therapeutic agent to combat breast cancer [29,30].

## 2. Results and Discussion

The optimization studies focused on the base-promoted reaction of *N*-benzyl-1-phenylmethanimine with each ester substrate in DMF at a series of temperatures to assess the minimum temperature required to give a reasonable conversion rate to the product. In the case of the nitro-activated phenylacetic ester (Table 1, EWG = NO_2_), the reaction was performed under aerobic conditions at 75 °C, 90 °C, and 105 °C. These experiments demonstrated that the reaction proceeded reasonably in 18–24 h at 90–95 °C and 12–16 h at 105–110 °C. Based on these observations, the reaction was performed at the lower temperature as this yielded a cleaner product. Similarly, temperatures between 75 and 130 °C were evaluated for the cyano-activated substrate (Table 1, EWG = CN). In this case, a significant reaction was not observed until 115–120 °C, and the final temperature utilized was 125–130 °C. Though our recent projects on analogous processes indicated that 2 equivalents of K_2_CO_3_ were sufficient for full conversion to the product, we explored reactions with 1 and 3 equivalents of base as well. Here, as in our past efforts, 2 equivalents of base gave the best result. Reactions were monitored by thin-layer chromatography and were completed in 12–24 h. Attempts to explore reactions with other less potent S_N_Ar activating groups such as trifluoromethyl (commercially available) in 1,3-dimethyl-3,4,5,6-tetrahydro-2(1*H*)-pyrimidinone (DMPU) required higher temperatures (>150 °C) but gave predominantly decomposition products.

The results of the current study are summarized in Table 1. Spectral data for each product is available in the Supplementary Materials. In the nitro series, the substrate cyclized with *N*-isobutyl-1-phenylmethanimine (expt. no. 3.2.1) as well as other imines derived from acetaldehyde (expt. nos. 3.2.2–3.2.5) and various benzaldehyde derivatives (expt. nos. 3.2.6–3.2.21). At the more elevated temperatures required for the cyano-activated precursor (>115 °C), primary alkyl amines and imines derived from these failed to give indole products under sealed tube conditions. Finally, it was noted that the alignment of the imine with the substrate for ring formation often required overcoming significant steric hindrance. Thus, we evaluated the use of phenethylamine instead of benzylamine to prepare some of the imines (expt. nos. 3.2.7, 3.2.13–3.2.15, and 3.2.17–3.2.21). The phenethyl group should be less sterically demanding than benzyl since the phenyl is further from the reaction site. Although we explored only a limited number of examples, we found that phenethylamine generally afforded superior yields to those observed with benzylamine for each ester. For example, in the nitro-activated substrate, the change from benzylamine (expt. no. 3.2.6) to phenethylamine (expt. no. 3.2.7) showed a minimal yield enhancement from 89% of **7** to 91% of **8** The corresponding cyano-activated precursor (expt. nos. 3.2.16 and 3.2.17) exhibited a greater yield differential increasing from 82% of **17** to 96% of **18**.

**Scheme 2:**
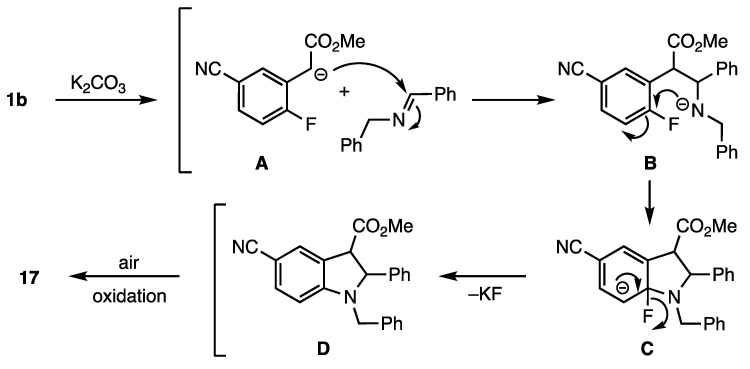
Presumed mechanism for the [3+2] approach to prepare methyl 1-benzyl-2-phenyl-5-cyano-1*H*-indole-3-carboxylate (**17**) from **1b**.

The presumed mechanism for the reaction is illustrated in Scheme 2. The imine pictured was derived from the condensation of benzaldehyde with benzylamine in DMF solution. This was best formed prior to addition of the ester and was not isolated. Attempts to accelerate this initial step with powdered 4 Å molecular sieves failed to improve the overall reaction outcome. Once prepared, the imine solution was added to one equivalent of ester and two equivalents of dry K_2_CO_3_ in anhydrous DMF and heat was applied. Following deprotonation of the ester to form anion A, addition to the polarized imine double bond and intramolecular cyclization of the resulting amine/anion B at the fluorine-bearing carbon of the aromatic ring would give Meisenheimer complex C. Finally, rearomatization with loss of KF would deliver indoline D (not observed) which would immediately air oxidize to the indole product.

## 3. Materials and Methods

### 3.1. General Methods

All reactions were performed under air in oven-dried glassware. All commercial reagents and solvents were used as received. Reactions were monitored by TLC on Miles Scientific No. P21521 silica gel GF plates (Miles Scientific, Newark, DE, USA). Preparative separations were performed on Davisil^®^, grade 62, 60–200 mesh silica gel containing 0.5% of UV-05 phosphor (both from Sorbent Technologies, Norcross, GA, USA) slurry packed into quartz columns. Band elution for all chromatographic separations was monitored using a hand-held UV light source (Fisher Scientific, Pittsburgh, PA, USA). Melting points (uncorrected) were obtained using a MEL-TEMP apparatus (Thomas Scientific, Cambridge, MA, USA). FT-IR spectra were obtained using an Agilent Cary 630 spectrometer (Agilent Technologies, Santa Clara, CA, USA) with ATR detection. ^1^H- and ^13^C-NMR spectra were measured using a Bruker Avance 400 system (Bruker, Billerica, MA, USA) at 400 MHz and 101 MHz, respectively, in CDCl_3_ containing 0.05% *v*/*v* tetramethylsilane as the internal standard at δ 0.00. ^19^F spectra were collected on the same instrument and are referenced to internal C_6_H_5_F at δ −113.15. Chemical shifts are given in δ (ppm) units relative to the standard and coupling constants (*J*) are given in Hz. Low-resolution mass spectra were obtained using an Advion expression^®^ single quadrupole compact mass spectrometer system using ESI positive ion mode at 3500 V (Advion, Ithaca, NY, USA). Elemental analyses (±0.4%) on all new compounds were performed by Atlantic Microlabs (Norcross, GA, USA).

### 3.2. Representative Procedure for Preparing 1,2,5-Trisubstituted 1H-Indole-3-carboxylate Esters

A solution of 1.17 mmol of the amine and 1.17 mmol of the aldehyde in 4 mL of dry DMF was stirred at 23 °C for 6 h. In a separate flask, 0.33 g (2.34 mmol, 2 equiv) of anhydrous K_2_CO_3_ was added to a solution of 0.25 g (1.17 mmol) of methyl 2-(2-fluoro-5-nitrophenyl)acetate in 4 mL of dry DMF at 23 °C. The imine solution was transferred by syringe to the deprotonated ester, and the mixture was stirred at 90 °C (silicone oil bath) for 12–24 h. The reaction was cooled, poured into water (50 mL) and extracted with ethyl acetate (3 × 50 mL). The combined ethyl acetate layers were washed with saturated NH_4_Cl (1 × 50 mL) and saturated NaCl (1 × 50 mL) and then dried (Na_2_SO_4_). The solution was concentrated under vacuum and purified by silica gel column chromatography using 15% ethyl acetate in hexane. The products were triturated with ether or ether-hexane mixtures. For compounds derived from methyl 2-(5-cyano-2-fluorophenyl)acetate (0.25 g, 1.29 mmol) and benzylamine (0.16 g, 1.29 mmol), the same procedure was used except that the cyclization required 12–24 h at 120 °C. The following compounds were prepared.

#### 3.2.1. Methyl 1-Isobutyl-5-nitro-2-phenyl-1*H*-indole-3-carboxylate (**2**)

Yield: 0.31 g (75%) as a white solid, m.p. 163–164 °C; IR: 1718 (C=O), 1528 (NO_2_), 1339 (NO_2_) cm^−1^; ^1^H NMR (400 MHz, CDCl_3_): δ 9.15 (d, *J* = 2.3 Hz, 1H, ArH), 8.21 (dd, *J* = 9.1, 2.3 Hz, 1H, ArH), 7.54–7.50 (complex, 3H, ArH), 7.45 (d, *J* = 9.1 Hz, 1H, ArH), 7.41–7.37 (complex, 2H, ArH), 3.89 (d, *J* = 7.7 Hz, 2H, CH_2_), 3.81 (s, 3H, CO_2_Me), 2.02 (nonet, *J* = 6.9 Hz, 1H, CH), 0.72 (d, *J* = 6.7 Hz, 6H, 2 Me); ^13^C NMR {^1^H} (101 MHz, CDCl_3_): δ 164.5 (C=O), 150.0, 143.4, 139.1, 130.5, 130.3, 129.5, 128.3, 125.9, 119.2, 118.3, 110.7, 107.4, 51.7 (ArOMe), 51.2 (CO_2_Me), 29.7 (CH), 28.9 (CH_2_), 20.0 (2 Me); MS (*m*/*z*) 353 (M + H)^+^; Anal. Calcd for C_20_H_20_N_2_O_4_: C, 68.17; H, 5.72; N, 7.95. Found: C, 68.12; H, 5.70; N, 7.91.

#### 3.2.2. Methyl 1-Benzyl-2-methyl-5-nitro-1*H*-indole-3-carboxylate (**3**)

Yield: 0.24 g (64%) as a yellow solid, m.p. 148–149 °C; IR: 1703 (C=O), 1519 (NO_2_), 1341 (NO_2_) cm^−1^; ^1^H NMR (400 MHz, CDCl_3_): δ 9.06 (s, *J* = 2.3 Hz, 1H, ArH), 8.10 (dd, *J* = 9.0, 2.3 Hz, 1H, ArH), 7.33–7.28 (complex, 4H, ArH), 6.96 (m, 2H, ArH), 5.42 (s, 2H, CH_2_), 4.00 (s, 3H, CO_2_Me), 2.78 (s, 3H, Me); ^13^C NMR {^1^H} (101 MHz, CDCl_3_): δ 165.5 (C=O), 148.5, 143.4, 139.2, 135.2, 129.2, 128.2, 126.0, 125.8, 118.6, 118.1, 109.7, 106.7, 51.3 (CO_2_Me), 47.1 (CH_2_), 12.1 (Me); MS (*m*/*z*) 325 (M + H)^+^; Anal. Calcd for C_18_H_16_N_2_O_4_: C, 66.66; H, 4.97; N, 8.64. Found: C, 66.61; H, 4.94; N, 8.58.

#### 3.2.3. Methyl 2-Methyl-1-(4-methylbenzyl)-5-nitro-1*H*-indole-3-carboxylate (**4**)

Yield: 0.27 g (68%) as a yellow solid, m.p. 161–162 °C; IR: 1703 (C=O), 1518 (NO_2_), 1340 (NO_2_) cm^−1^; ^1^H NMR (400 MHz, CDCl_3_): δ 9.05 (d, *J* = 2.3 Hz, 1H, ArH), 8.10 (dd, *J* = 9.0, 2.3 Hz, 1H, ArH), 7.29 (d, *J* = 9.0 Hz, 1H, ArH), 7.11 (d, *J* = 7.9 Hz, 2H, ArH), 6.85 (d, *J* = 7.9 Hz, 2H, ArH), 5.37 (s, 2H, CH_2_), 4.00 (s, 3H, CO_2_Me), 2.78 (s, 3H, Me), 2.31 (s, 3H, Me); ^13^C NMR {^1^H} (101 MHz, CDCl_3_): δ 165.5 (C=O), 148.6, 143.4, 139.2, 138.0, 132.1, 129.9, 126.0, 125.8, 118.6, 118.1, 109.7, 106.6, 51.3 (CO_2_Me), 46.9 (CH_2_), 21.1 (Me), 12.1 (Me); MS (*m*/*z*) 339 (M + H)^+^; Anal. Calcd for C_19_H_18_N_2_O_4_: C, 67.45; H, 5.36; N, 8.28. Found: C, 67.41; H, 5.35; N, 8.21.

#### 3.2.4. Methyl 1-(4-Chlorobenzyl)-1-methyl-5-nitro-1*H*-indole-3-carboxylate (**5**)

Yield: 0.36 g (85%) as a white solid, m.p. 191–192 °C; IR: 1705 (C=O), 1522 (NO_2_), 1336 (NO_2_) cm^−1^; ^1^H NMR (400 MHz, CDCl_3_): δ 9.06 (d, *J* = 2.3 Hz, 1H, ArH), 8.11 (dd, *J* = 9.0, 2.3 Hz, 1H, ArH), 7.29 (d, *J* = 8.5 Hz, 2H, ArH), 7.26 (obscured, 1H, ArH), 6.90 (d, *J* = 8.5 Hz, 2H, ArH), 5.39 (s, 2H, CH_2_), 4.01 (s, 3H, CO_2_Me), 2.77 (s, 3H, Me); ^13^C NMR {^1^H} (101 MHz, CDCl_3_): δ 165.4 (C=O), 148.2, 143.5, 139.0, 134.2, 133.7, 129.5, 127.2, 126.1, 118.7, 118.3, 109.5, 106.9, 51.4 (CO_2_Me), 46.5 (CH_2_), 12.1 (Me); MS (*m*/*z*) 359, 361 (ca. 3:1, (M + H)^+^); Anal. Calcd for C_18_H_15_ClN_2_O_4_: C, 60.26; H, 4.21; N, 7.81. Found: C, 60.14; H, 4.18; N, 7.69.

#### 3.2.5. Methyl 1-(2-Chlorobenzyl)-2-methyl-5-nitro-1*H*-indole-3-carboxylate (**6**)

Yield: 0.25 g (60%) as a yellow solid, m.p. 167–168 °C; IR: 1710 (C=O), 1519 (NO_2_), 1341 (NO_2_) cm^−1^; ^1^H NMR (400 MHz, CDCl_3_): δ 9.07 (d, *J* = 2.3 Hz, 1H, ArH), 8.10 (dd, *J* = 9.0, 2.3 Hz, 1H, ArH), 7.47 (dd, *J* = 8.0, 1.2 Hz, 1H, ArH), 7.28–7.23 (complex, 1H, ArH), 7.23 (d, *J* = 6.8 Hz, 1H, ArH), 7.08 (td, *J* = 7.7, 1.3 Hz, 1H, ArH), 6.25 (dd, *J* = 7.7, 1.5 Hz, 1H, ArH), 5.48 (s, 2H, CH_2_), 4.02 (s, 3H, CO_2_Me), 2.75 (s, 3H, Me); ^13^C NMR {^1^H} (101 MHz, CDCl_3_): δ 165.4 (C=O), 148.5, 143.6, 139.1, 132.7, 132.1, 129.9, 129.4, 127.6, 126.2, 126.1, 118.7, 118.3, 109.6, 106.9, 51.4 (CO_2_Me), 44.9 (CH_2_), 11.9 (Me); MS (*m*/*z*) 359, 361 (ca. 3:1, (M + H)^+^); Anal. Calcd for C_18_H_15_ClN_2_O_4_: C, 60.26; H, 4.21; N, 7.81. Found: C, 60.22; H, 4.19; N, 7.77.

#### 3.2.6. Methyl 1-Benzyl-5-nitro-2-phenyl-1*H*-indole-3-carboxylate (**7**)

Yield: 0.40 g (89%) as a brick-red solid, m.p. 179–180 °C; IR: 1701 (C=O), 1516 (NO_2_), 1337 (NO_2_) cm^−1^; ^1^H NMR (400 MHz, CDCl_3_): δ 9.17 (d, *J* = 2.3 Hz, 1H, ArH), 8.12 (dd, *J* = 9.0, 2.3 Hz, 1H, ArH), 7.52–7.42 (complex, 3H, ArH), 7.36 (d, *J* = 7.9 Hz, 2H, ArH), 7.28–7.24 (complex, 4H, ArH), 6.89 (m, 2H, ArH), 5.24 (s, 2H, CH_2_), 3.83 (s, 3H, CO_2_Me); ^13^C NMR {^1^H} (101 MHz, CDCl_3_): δ 164.4 (C=O), 150.1, 143.6, 139.1, 135.8, 130.0, 129.8, 129.0, 128.4, 128.0, 126.2, 125.9, 119.2, 118.7, 111.0, 107.6, 51.3 (CO_2_Me), 48.0 (CH_2_) (one aromatic carbon unresolved); MS (*m*/*z*) 387 (M + H)^+^; Anal. Calcd for C_23_H_18_N_2_O_4_: C, 71.49; H, 4.70; N, 7.25. Found: C, 71.45; H, 4.69; N, 7.20.

#### 3.2.7. Methyl 5-Nitro-1-phenethyl-2-phenyl-1*H*-indole-3-carboxylate (**8**)

Yield: 0.43 g (91%) as a brick-red solid, m.p. 175–176 °C; IR: 1709 (C=O), 1517 (NO_2_), 1340 (NO_2_) cm^−1^; ^1^H NMR (400 MHz, CDCl_3_): δ 9.15 (d, *J* = 2.3 Hz, 1H, ArH), 8.19 (dd, *J* = 9.0, 2.3 Hz, 1H, ArH), 7.54–7.45 (complex, 3H, ArH), 7.39 (d, *J* = 9.0 Hz, 1H, ArH), 7.20–7.15 (complex, 5H, ArH), 6.78 (m, 2H, ArH), 4.24 (t, *J* = 7.4 Hz, 2H, CH_2_), 3.79 (s, 3H, CO_2_Me), 2.92 (t, *J* = 7.4 Hz, 2H, CH_2_); ^13^C NMR {^1^H} (101 MHz, CDCl_3_): δ 164.4 (C=O), 149.8, 143,4, 138.6, 136.9, 130.2, 129.8, 129.5, 128.8, 128.6, 128.3, 127.1, 126.0, 119.3, 118.4, 110.2, 107.5, 51.2 (CO_2_Me), 46.0 (CH_2_), 36.0 (CH_2_); MS (*m*/*z*) 401 (M + H)^+^; Anal. Calcd for C_24_H_20_N_2_O_4_: C, 66.66; H, 4.97; N, 8.64. Found: C, 66.58; H, 4.93; N, 8.55.

#### 3.2.8. Methyl 1-(4-Methylbenzyl)-5-nitro-2-phenyl-1*H*-indole-3-carboxylate (**9**)

Yield: 0.41 g (81%) as a light yellow solid, m.p. 100–101 °C; IR: 1713 (C=O), 1517 (NO_2_), 1342 (NO_2_) cm^−1^; ^1^H NMR (400 MHz, CDCl_3_): δ 9.16 (d, *J* = 2.3 Hz, 1H, ArH), 8.12 (dd, *J* = 9.0, 2.3 Hz, 1H, ArH), 7.52–7.43 (complex, 3H, ArH), 7.37 (m, 2H, ArH), 7.27 (obscured, 1H, ArH), 7.06 (d, *J* = 7.8 Hz, 2H, ArH), 6.78 (d, *J* = 7.8 Hz, 2H, ArH), 5.20 (s, 2H, CH_2_), 3.83 (s, 3H, CO_2_Me), 2.30 (s, 3H, Me); ^13^C NMR {^1^H} (101 MHz, CDCl_3_): δ 164.5 (C=O), 150.1, 143.6, 139.1, 137.8, 132.7, 130.02, 130.00, 129.72, 129.66, 128.4, 126.2, 125.9, 119.2, 118.6, 111.1, 107.5, 51.3 (CO_2_Me), 47.9 (CH_2_), 21.1 (Me); MS (*m*/*z*) 401 (M + H)^+^; Anal. Calcd for C_24_H_20_N_2_O_4_: C, 66.66; H, 4.97; N, 8.64. Found: C, 66.57; H, 4.95; N, 8.59.

#### 3.2.9. Methyl 1-Benzyl-2-(4-fluorophenyl)-5-nitro-1*H*-indole-3-carboxylate (**10**)

Yield: 0.35 g (64%) as a white solid, m.p. 165–166 °C; IR: 1714 (C=O), 1521 (NO_2_), 1343 (NO_2_) cm^−1^; ^1^H NMR (400 MHz, CDCl_3_): δ 9.16 (d, *J* = 2.3 Hz, 1H, ArH), 8.14 (dd, *J* = 9.0, 2.3 Hz, 1H, ArH), 7.36– 7.24 (complex, 6H, ArH), 7.15 (t, *J* = 8.5 Hz, 2H, ArH), 6.87 (m, 2H, ArH), 5.24 (s, 2H, CH_2_), 3.84 (s, 3H, CO_2_Me); ^13^C NMR {^1^H} (101 MHz, CDCl_3_): δ 164.4 (C=O), 153.5, (d, *J* = 250.4 Hz, ArC-F), 148.9, 143.7, 139.1, 135.7, 132.1 (d, *J* = 8.5 Hz), 129.1, 128.1, 126.0, 125.9 (d, *J* = 3.7 Hz), 125.8, 119.3, 118.8, 115.6 (d, *J* = 21.9 Hz), 111.0, 108.0, 51.4 (CO_2_Me), 48.0 (CH_2_); ^19^F NMR {^1^H} (376 MHz, CDCl_3_ referenced to C_6_H_5_F): δ –110.49; MS (*m*/*z*) 405 (M + H)^+^; Anal. Calcd for C_23_H_17_FN_2_O_4_: C, 68.31; H, 4.24; N, 6.93. Found: C, 68.27; H, 4.23; N, 6.88.

#### 3.2.10. Methyl 1-Benzyl-2-(4-chlorophenyl)-5-nitro-1*H*-indole-3-carboxylate (**11**)

Yield: 0.43 g (85%) as a white solid, m.p. 110–111 °C; IR: 1705 (C=O), 1518 (NO_2_), 1340 (NO_2_) cm^−1^; ^1^H NMR (400 MHz, CDCl_3_): δ 9.17 (d, *J* = 2.3 Hz, 1H, ArH), 8.15 (dd, *J* = 9.0, 2.3 Hz, 1H, ArH), 7.54–7.43 (complex, 3H, ArH), 7.34 (m, 2H, ArH), 7.26 (obscured, 1H, ArH), 7.23 (d, *J* = 8.5 Hz, 2H, ArH), 6.80 (d, *J* = 8.5 Hz, 2H, ArH), 5.21 (s, 2H, CH_2_), 3.83 (s, 3H, CO_2_Me); ^13^C NMR {^1^H} (101 MHz, CDCl_3_): δ 164.3 (C=O), 149.8, 143.7, 138.9, 134.3, 133.9, 129.91, 129.86, 129.8, 129.2, 128.5, 127.3, 126.2, 119.3, 118.8, 110.7, 107.9, 51.4 (CO_2_Me), 47.4 (CH_2_); MS (*m*/*z*) 421, 423 (ca. 3:1, (M + H)^+^); Anal. Calcd for C_23_H_17_ClN_2_O_4_: C, 65.64; H, 4.07; N, 6.66. Found: C, 65.65; H, 4.05; N, 6.57.

#### 3.2.11. Methyl 1-Benzyl-2-(2-chlorophenyl)-5-nitro-1*H*-indole-3-carboxylate (**12**)

Yield: 0.29 g (59%) as a white solid, m.p. 179–180 °C; IR: 1716 (C=O), 1519 (NO_2_), 1340 (NO_2_) cm^−1^; ^1^H NMR (400 MHz, CDCl_3_): δ 9.18 (d, *J* = 2.3 Hz, 1H, ArH), 8.15 (dd, *J* = 9.0, 2.3 Hz, 1H, ArH), 7.54 (dd, *J* = 8.2, 1.3 Hz, 1H, ArH), 7.46 (td, *J* = 7.4, 1.8 Hz, 1H, ArH), 7.35–7.30 (complex, 2H, ArH), 7.26–7.22 (complex, 4H, ArH), 6.89 (m, 2H, ArH), 5.30 (d, *J* = 16.4 Hz, 1H, H_a_ of CH_2_), 5.09 (d, *J* = 16.4 Hz, 1H, H_b_ of CH_2_), 3.83 (s, 3H, CO_2_Me); ^13^C NMR {^1^H} (101 MHz, CDCl_3_): δ 164.1 (C=O), 146.6, 143.6, 139.1, 135.4, 134.4, 131.9, 131.2, 129.7, 129.6, 128.9, 128.1, 126.8, 126.3, 125.9, 119.3, 118.8, 111.1, 108.6, 51.4 (CO_2_Me), 48.3 (CH_2_); MS (*m*/*z*) 421, 423 (ca. 3:1, (M + H)^+^); Anal. Calcd for C_23_H_17_ClN_2_O_4_: C, 65.64; H, 4.07; N, 6.66. Found: C, 65.56; H, 4.03; N, 6.54.

#### 3.2.12. Methyl 1-Benzyl-2-(3-methoxyphenyl)-5-nitro-1*H*-indole-3-carboxylate (**13**)

Yield: 0.30 g (61%) as a yellow solid, m.p. 275–276 °C; IR: 2844 (OMe), 1713 (C=O), 1524 (NO_2_), 1346 (NO_2_) cm^−1^; ^1^H NMR (400 MHz, CDCl_3_): δ 9.17 (d, *J* = 2.3 Hz, 1H, ArH), 8.13 (dd, *J* = 9.0, 2.3 Hz, 1H, ArH), 7.37 (dd, *J* = 8.3, 7.5 Hz, 1H, ArH), 7.29–7.24 (complex, 4H, ArH), 7.02 (ddd, *J* = 8.4, 2.6, 1.0 Hz, 1H, ArH), 6.95 (dt, *J* = 7.6, 1.3 Hz, 1H, ArH), 6.91–6.87 (complex, 2H, ArH), 6.86 (dd, *J* = 2.6, 1.5 Hz, 1H, ArH), 5.24 (s, 2H, CH_2_), 3.85 (s, 3H), 3.68 (s, 3H) (ArOMe and CO_2_Me); ^13^C NMR {^1^H} (101 MHz, CDCl_3_): δ 164.4 (C=O), 159.3, 149.8, 143.7, 139.1, 135.9, 131.1, 129.5, 129.0, 128.7, 128.0, 127.5, 126.1, 125.9, 122.3, 119.3, 118.7, 115.7, 115.2, 110.9, 107.6, 55.1 (ArOMe), 51.3 (CO_2_Me), 48.1 (CH_2_); MS (*m*/*z*) 417 (M + H)^+^; Anal. Calcd for C_24_H_20_N_2_O_5_: C, 69.22; H, 4.84; N, 6.78. Found: C, 69.17; H, 4.81; N, 6.71.

#### 3.2.13. Methyl 2-(4-Methylphenyl)-5-nitro-1-phenethyl-1*H*-indole-3-carboxylate (**14**)

Yield: 0.44 g (90%) as a white solid, m.p. 169–171 °C; IR: 1712 (C=O), 1518 (NO_2_), 1338 (NO_2_) cm^−1^; ^1^H NMR (400 MHz, CDCl_3_): δ 9.12 (d, *J* = 2.3 Hz, 1H, ArH), 8.17 (dd, *J* = 9.0, 2.3 Hz, 1H, ArH), 7.37 (d, *J* = 9.0 Hz, 1H, ArH), 7.28 (d, *J* = 7.7 Hz, 2H, ArH), 7.22–7.15 (complex, 3H, ArH), 7.08 (d, *J* = 7.7 Hz, 2H, ArH), 6.82–6.78 (complex, 2H, ArH), 4.25 (t, *J* = 7.4 Hz, 2H, CH_2_), 3.81 (s, 3H, CO_2_Me), 2.92 (t, *J* = 7.4 Hz, 2H, CH_2_), 2.46 (s, 3H, Me); ^13^C NMR {^1^H} (101 MHz, CDCl_3_): δ 164.5 (C=O), 150.1, 143.4, 139.6, 138.6, 137.0, 129.7, 129.1, 128.8, 128.6, 127.14, 127.07, 126.0, 119.2, 118.3, 110.2, 107.4, 51.2 (CO_2_Me), 45.9 (CH_2_), 36.0 (CH_2_), 21.5 (Me); MS (*m*/*z*) 415 (M + H)^+^; Anal. Calcd for C_25_H_22_N_2_O_4_: C, 72.43; H, 5.35; N, 6.76. Found: C, 72.38; H, 5.31; N, 6.73.

#### 3.2.14. Methyl 2-(4-Fluorophenyl)-5-nitro-1-phenethyl-1*H*-indole-3-carboxylate (**15**)

Yield: 0.41 g (64%) as a white solid, m.p. 189–190 °C; IR: 1722 (C=O), 1520 (NO_2_), 1344 (NO_2_) cm^−1^; ^1^H NMR (400 MHz, CDCl_3_): δ 9.14 (d, *J* = 2.3 Hz, 1H, ArH), 8.21 (dd, *J* = 9.0, 2.3 Hz, 1H, ArH), 7.42 (d, *J* = 9.0 Hz, 1H, ArH), 7.23–7.09 (complex, 5H, ArH), 7.06 (m, 2H, ArH), 6.74 (d, *J* = 7.8 Hz, 2H, ArH), 4.25 (t, *J* = 7.1 Hz, 2H, CH_2_), 3.80 (s, 3H, CO_2_Me), 2.94 (t, *J* = 7.1 Hz, 2H, CH_2_); ^13^C NMR {^1^H} (101 MHz, CDCl_3_): δ 164.4 (C=O), 163.2 (d, *J* = 231.0 Hz, ArC–F), 148.7, 143.5, 138.5, 136.9, 131.9 (d, *J* = 8.5 Hz, 128.9, 128.6, 127.2, 126.1 (d, *J* = 3.4 Hz), 125.9, 119.3, 118.6, 115.5 (d, *J* = 21.9 Hz), 110.4, 107.7, 51.7 (CO_2_Me), 46.0 (CH_2_), 35.8 (CH_2_); ^19^F NMR {^1^H} (376 MHz, CDCl_3_ referenced to C_6_H_5_F): δ –110.93; MS (*m*/*z*) 419 (M + H)^+^; Anal. Calcd for C_24_H_19_FN_2_O_4_: C, 68.89; H, 4.58; N, 6.70. Found: C, 68.85; H, 4.57; N, 6.63.

#### 3.2.15. Methyl 2-(4-Methoxyphenyl)-5-nitro-1-phenethyl-1*H*-indole-3-carboxylate (**16**)

Yield: 0.43 g (85%) as a light yellow solid, m.p. 174–175 °C; IR: 1717 (C=O), 1519 (NO_2_), 1342 (NO_2_) cm^−1^; ^1^H NMR (400 MHz, CDCl_3_): δ 9.12 (d, *J* = 2.3 Hz, 1H, ArH), 8.18 (dd, *J* = 9.0, 2.3 Hz, 1H, ArH), 7.37 (d, *J* = 9.0 Hz, 1H, ArH), 7.23–7.15 (complex, 3H, ArH), 7.10 (d, *J* = 8.9 Hz, 2H, ArH), 6.98 (d, *J* = 8.9 Hz, 2H, ArH), 6.81 (m, 2H, ArH), 4.26 (t, *J* = 7.3 Hz, 2H, CH_2_), 3.90 (s, 3H), 3.81 (s, 3H) (ArOMe and CO_2_Me), 2.92 (t, *J* = 7.3 Hz, 2H, CH_2_); ^13^C NMR {^1^H} (101 MHz, CDCl_3_): δ 164.5 (C=O), 160.4, 149.9, 143.4, 138.6, 137.0, 131.2, 128.8, 128.6, 127.1, 126.0, 122.1, 119.2, 118, 113.8, 110.2, 107.4, 55.4 (ArOMe), 51.2 (CO_2_Me), 45.9 (CH_2_), 35.9 (CH_2_); MS (*m*/*z*) 431 (M + H)^+^; Anal. Calcd for C_25_H_22_N_2_O_5_: C, 69.76; H, 5.15; N, 6.51. Found: C, 69.69; H, 5.14; N, 6.42.

#### 3.2.16. Methyl 1-Benzyl-5-cyano-2-phenyl-1*H*-indole-3-carboxylate (**17**)

Yield: 0.39 g (82%) as a white solid, m.p. 190–192 °C; IR: 2232 (C≡N), 1703 (C=O) cm^−1^; ^1^H NMR (400 MHz, CDCl_3_): δ 8.63 (dd, *J* = 1.6, 0.7 Hz, 1H, ArH), 7.53–7.43 (complex, 4H, ArH), 7.37–7.34 (complex, 2H, ArH), 7.29–7.23 (complex, 3H, ArH), 6.87 (m, 2H, ArH), 5.22 (s, 2H, CH_2_), 3.80 (s, 3H, CO_2_Me); ^13^C NMR {^1^H} (101 MHz, CDCl_3_): δ 164.6 (C=O), 149.3, 137.9, 135.9, 130.1, 130.0, 129.7, 129.0, 128.3, 127.9, 127.8, 126.5, 126.1, 125.9, 120.3 (C≡N), 111.8, 106.4, 105.5, 51.2 (CO_2_Me), 47.9 (CH_2_); MS (*m*/*z*) 367 (M + H)^+^; Anal. Calcd for C_24_H_18_N_2_O_2_: C, 78.67; H, 4.95; N, 8.73. Found: C, 78.61; H, 4.95; N, 8.69.

#### 3.2.17. Methyl 5-Cyano-1-phenethyl-2-phenyl-1*H*-indole-3-carboxylate (**18**)

Yield: 0.47 g (96%) as a white solid, m.p. 183–184 °C; IR: 2234 (C≡N), 1700 (C=O) cm^−1^; ^1^H NMR (400 MHz, CDCl_3_): δ 8.60 (d, *J* = 1.6 Hz, 1H, ArH), 7.55–7.44 (complex, 4H, ArH), 7.41 (d, *J* = 8.6 Hz, 1H, ArH), 7.17 (complex, 5H, ArH), 6.77 (m, 2H, ArH), 4.21 (t, *J* = 7.4 Hz, 2H, CH_2_), 3.76 (s, 3H, CO_2_Me), 2.90 (t, *J* = 7.4 Hz, 2H, CH_2_); ^13^C NMR {^1^H} (101 MHz, CDCl_3_): δ 166.6 (C=O), 148.9, 137.4, 137.0 (Ar), 130.3, 129.8, 129.4, 128.8, 128.6, 128.3, 127.8, 127.0, 126.4, 125.8, 120.4 (C≡N), 111.0, 106.2, 105.3, 51.1 (CO_2_Me), 45.8 (CH_2_), 36.0 (CH_2_); MS (*m*/*z*) 381 (M + H)^+^; Anal. Calcd for C_25_H_20_N_2_O_2_: C, 78.93; H, 5.30; N, 7.36. Found: C, 78.89; H, 5.28; N, 7.28.

#### 3.2.18. Methyl 5-Cyano-2-(4-methylphenyl)-1-phenethyl-1*H*-indole-3-carboxylate (**19**)

Yield: 0.43 g (85%) as a white solid, m.p. 189–190 °C; IR: 2225 (C≡N), 1698 (C=O) cm^−1^; ^1^H NMR (400 MHz, CDCl_3_): δ 8.58 (d, *J* = 1.6 Hz, 1H, ArH), 7.51 (dd, *J* = 8.5, 1.6 Hz, 1H, ArH), 7.39 (d, *J* = 8.5 Hz, 1H, ArH), 7.27 (d, *J* = 8.1 Hz, 2H, ArH), 7.21–7.15 (complex, 3H, ArH), 7.06 (d, *J* = 8.1 Hz, 2H, ArH), 6.80 (m, 2H, ArH), 4.22 (t, *J* = 7.4 Hz, 2H, CH_2_), 3.78 (s, 3H, CO_2_Me), 2.90 (t, *J* = 7.4 Hz, 2H, CH_2_), 2.45 (s, 3H, Me); ^13^C NMR {^1^H} (101 MHz, CDCl_3_): δ 164.6 (C=O), 149.3, 139.5, 137.4 (Ar), 137.1, 129., 129.1, 128.8, 128.6, 127.8, 127.2, 127.0, 126.4, 125.7, 120.4 (C≡N), 111.0, 106.1, 105.2, 51.1 (CO_2_Me), 45.8 (CH_2_), 36.0 (CH_2_), 21.5 (Me); MS (*m*/*z*) 395 (M + H)^+^; Anal. Calcd for C_26_H_22_N_2_O_2_: C, 79.17; H, 5.62; N, 7.10. Found: C, 79.11; H, 5.59; N, 7.04.

#### 3.2.19. Methyl 5-Cyano-2-(4-fluorophenyl)-1-phenethyl-1*H*-indole-3-carboxylate (**20**)

Yield: 0.40 g (78%) as a white solid, m.p. 202–203 °C; IR: 2209 (C≡N), 1699 (C=O) cm^−1^; ^1^H NMR (400 MHz, CDCl_3_): δ 8.60 (d, *J* = 1.6 Hz, 1H, ArH), 7.55 (dd, *J* = 8.5, 1.6 Hz, 1H, ArH), 7.44 (d, *J* = 8.5 Hz, 1H, ArH), 7.25–7.15 (complex, 3H, ArH), 7.11 (t, *J* = 8.5 Hz, 2H, ArH), 7.05 (m, 2H, ArH), 6.74 (m, 2H, ArH), 4.22 (t, *J* = 7.2 Hz, 2H, CH_2_), 3.77 (s, 3H, CO_2_Me), 2.92 (t, *J* = 7.2 Hz, 2H, CH_2_); ^13^C NMR {^1^H} (101 MHz, CDCl_3_): δ 164.5 (C=O), 163.3 (d, *J* = 249.9 Hz, ArC–F), 147.9, 137.3, 137.0, 131.9 (d, *J* = 8.4 Hz), 128.9, 128.6, 127.9, 127.1, 126.3, 126.2 (d, *J* = 3.5 Hz), 125.9, 120.3 (C≡N), 115.5 (d, *J* = 21.7 Hz, 111.1, 106.4, 105.4, 51.2 (CO_2_Me), 45.8 (CH_2_), 35.8 (CH_2_); ^19^F NMR {^1^H} (376 MHz, CDCl_3_ referenced to C_6_H_5_F): δ –111.11; MS (*m*/*z*) 399 (M + H)^+^; Anal. Calcd for C_25_H_19_FN_2_O_2_: C, 75.36; H, 4.81; N, 7.03. Found: C, 75.32; H, 4.79; N, 6.97.

#### 3.2.20. Methyl 2-(4-Chlorophenyl)-5-cyano-1-phenethyl-1*H*-indole-3-carboxylate (**21**)

Yield: 0.38 g (78%) as a white solid, m.p. 164–165 °C; IR: 2231 (C≡N), 1700 (C=O) cm^−1^; ^1^H NMR (400 MHz, CDCl_3_): δ 8.61 (d, *J* = 1.6 Hz, 1H, ArH), 7.56 (dd, *J* = 8.1, 1.3 Hz, 1H, ArH), 7.52 (dd, *J* = 8.6, 1.6 Hz, 1H, ArH), 7.47 (td, *J* = 7.5, 1.6 Hz, 1H, ArH), 7.38 (d, *J* = 8.5 Hz, 1H, ArH), 7.31 (td, *J* = 7.6, 1.3 Hz, 1H, ArH), 7.21–7.14 (complex, 3H, ArH), 6.95 (dd, *J* = 7.6, 1.6 Hz, 1H, ArH), 6.79 (m, 2H, ArH), 4.29 (ddd, *J* = 14.1, 7.9, 5.2 Hz, 1H, H_a_ of CH_2_), 4.03 (dt, *J* = 15.6, 8.1 Hz, 1H, H_b_ of CH_2_), 3.77 (s, 3H, CO_2_Me), 2.91 (m, 2H, CH_2_); ^13^C NMR {^1^H} (101 MHz, CDCl_3_): δ 164.2 (C=O), 137.9, 137.4, 137.1, 134.1, 131.0, 129.6, 128.8, 128.7, 127.9, 127.1, 126.8, 126.1, 125.9, 120.3 (C≡N), 111.1, 106.8, 105.3, 51.2 (CO_2_Me), 46.1 (CH_2_), 35.9 (CH_2_); MS (*m*/*z*) 415, 417 (ca. 3:1, (M + H)^+^); Anal. Calcd for C_25_H_19_ClN_2_O_2_: C, 72.37; H, 4.62; N, 6.75. Found: C, 72.32; H, 4.61; N, 6.68.

#### 3.2.21. Methyl 5-Cyano-2-(4-methoxyphenyl)-1-phenethyl-1*H*-indole-3-carboxyl-ate (**22**)

Yield: 0.40 g (80%) as a white solid, m.p. 182–183 °C; IR: 2839 (OMe), 2229 (C≡N), 1698 (C=O) cm^−1^; ^1^H NMR (400 MHz, CDCl_3_): δ 8.58 (dd, *J* = 1.6, 0.7 Hz, 1H, ArH), 7.53 (dd, *J* = 7.0, 1.6 Hz, 1H, ArH), 7.40 (dd, *J* = 8.5, 0.7 Hz, 1H, ArH), 7.22–7.15 (complex, 3H, ArH), 7.09 (d, *J* = 8.7 Hz, 2H, ArH), 6.98 (d, *J* = 8.7 Hz, 2H, ArH), 6.79 (m, 2H, ArH), 4.23 (t, *J* = 7.4 Hz, 2H, CH_2_), 3.89 (s, 3H), 3.78 (s, 3H) (ArOMe and CO_2_Me), 2.91 (t, *J* = 7.4 Hz, 2H, CH_2_); ^13^C NMR {^1^H} (101 MHz, CDCl_3_): δ 164.7 (C=O), 160.3, 149.0, 137.4, 137.1, 131.2, 128.8, 128.6, 127.8, 127.0, 126.4, 125.7, 122.2, 120.4 (C≡N), 113.8, 111.0, 106.1, 105.2, 55.3 (ArOMe), 51.1 (CO_2_Me), 45.7 (CH_2_), 35.9 (CH_2_); MS (*m*/*z*) 411 (M + H)^+^; Anal. Calcd for C_26_H_22_N_2_O_3_: C, 76.08; H, 5.40; N, 6.82. Found: C, 76.01; H, 5.38; N, 6.74.

## 4. Conclusions

A new approach to 1,2,5-trisubstituted 1*H*-indole-3-carboxylic esters has been developed and studied. The method involves the reaction of an aldehyde-derived imine (2-atom reactant) with the K_2_CO_3_-derived anion from methyl 2-(2-fluoro-5-nitrophenyl)acetate or methyl 2-(5-cyano-2-fluorophenyl)acetate, each of which incorporates a 3-atom nucleophilic-electrophilic subunit. Heating the reaction initiates the [3+2] cyclization by addition of the anion to the imine followed by ring closure of the nitrogen of the adduct to the aromatic moiety by an S_N_Ar process. The scope of the reaction was somewhat narrowed by the fact that only highly activated 2-fluorophenylacetic esters can be used at temperatures ranging from 90–95 °C (NO_2_) to 125–130 °C (CN). Though atom efficient, steric hindrance in the reacting partners appears to limit the reaction. To reduce the steric hindrance in the reaction, phenethylamine was substituted for benzylamine in a number of reactions. This was found to afford minimal improvement to the yield in the nitro-activated substrates and moderate yield enhancement for cyano-activated esters. In all cases, the initial indoline formed was not observed but instead underwent spontaneous air oxidation to the give the aromatic heterocycle. Nonaromatic primary amines and aldehydes generated indoles with nitro-activated substrates but failed at the higher temperatures required for the cyano-activated precursors.

## Figures and Tables

**Table 1:** Reaction of imines with 2-(2-fluorophenyl)acetic esters **1a** and **1b** to prepare 1,2,5-trisubstituted 1*H*-indole-3-carboxylic esters **2**–**22**.

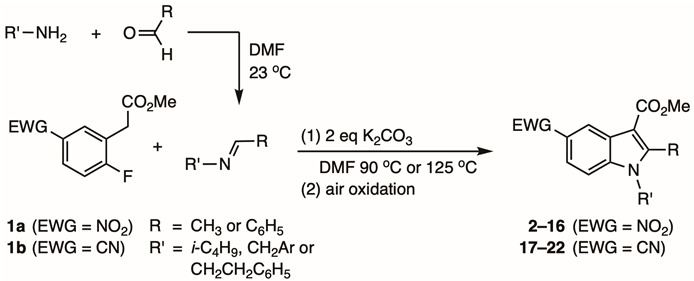					
Expt No.^a^	Methyl (2-Fluoro-phenyl)acetate Substrate	Aldehyde	Amine	Product	Yield (%)
3.2.1	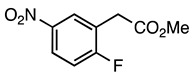 **1a**	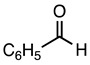	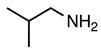	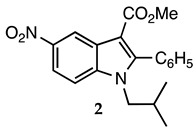	75
3.2.2	**1a**	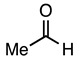	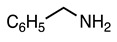	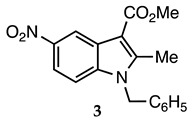	64
3.2.3	**1a**	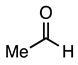	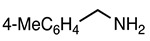	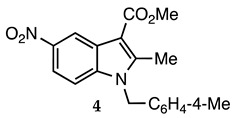	68
3.2.4	**1a**	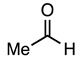	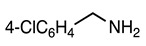	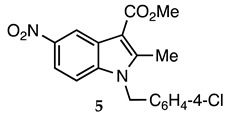	85
3.2.5	**1a**	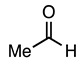	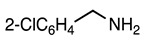	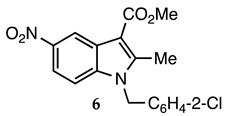	60
3.2.6	**1a**	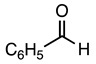	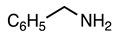	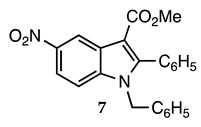	89
3.2.7	**1a**	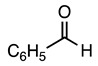	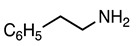	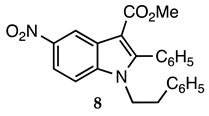	91
3.2.8	**1a**	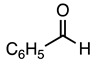	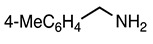	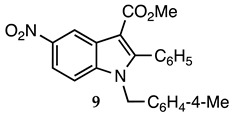	81
3.2.9	**1a**	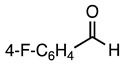	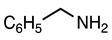	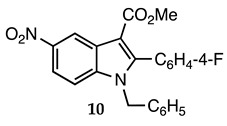	64
3.2.10	**1a**	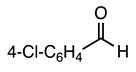	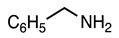	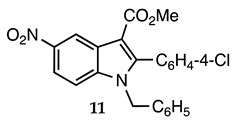	85
3.2.11	**1a**	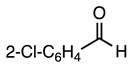	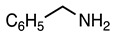	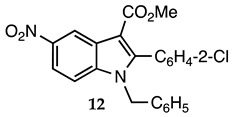	59
3.2.12	**1a**	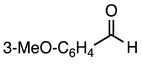	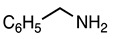	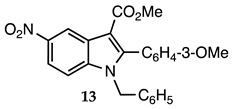	61
3.2.13	**1a**	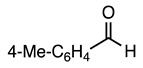	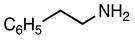	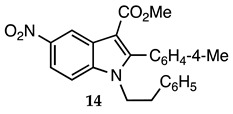	90
3.2.14	**1a**	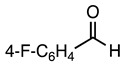	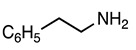	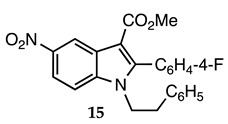	64
3.2.15	**1a**	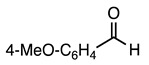	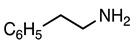	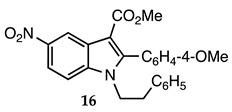	85
3.2.16	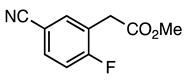 **1b**	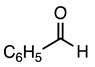	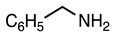	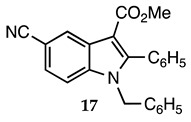	82
3.2.17	**1b**	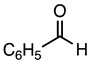	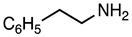	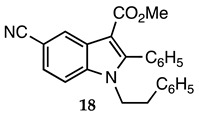	96
3.2.18	**1b**	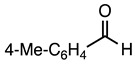	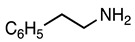	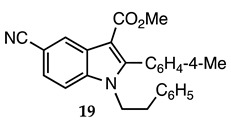	85
3.2.19	**1b**	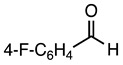	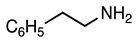	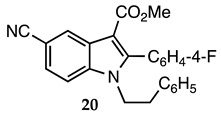	78
3.2.20	**1b**	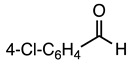	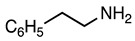	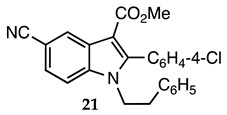	78
3.2.21	**1b**	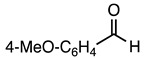	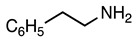	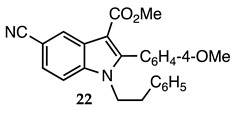	80

^a^ All reactions were run using 1.17 mmol of the aldehyde, 1.17 mmol of the amine, 1.17 mmol of the 2-(2-fluoro)phenylacetic ester, and 2.34 mmol of K_2_CO_3_ in DMF at 90–95 °C for the nitro-activated and 125–130 °C for the cyano-activated substrates.

## Data Availability

The original contributions presented in this study are included in the article/Supplementary Materials. Further inquiries can be directed to the corresponding author.

## Supplementary Materials

The following supporting information can be downloaded at: https://www.mdpi.com/article/10.3390/molecules30030444/s1. Copies of ^1^H-NMR, ^13^C-NMR, and where appropriate, ^19^F-NMR spectra for all cyclization products are provided.
