# Supplementary material for: Domino Synthesis of 1,2,5-Trisubstituted 1H-Indole-3-carboxylic Esters Using a [3+2] Strategy
Source: Molecules. 2025 Jan 21;30(3):444. doi: 10.3390/molecules30030444 (PMC11820459; doi:10.3390/molecules30030444)
Supplement: Supplementary file 1 [file molecules-30-00444-s001.zip › molecules-3397026-supplementary.pdf]

Domino Synthesis of 1,2,5-Trisubstituted 1H-Indole-3-carboxylic Esters Using a [3+2] Strategy

Siddhartha Maji , Kwabena Fobi, Ebenezer Ametsetor and Richard A. Bunce *

Department of Chemistry, Oklahoma State University, Stillwater, OK 74078-3071, USA; smaji@okstate.edu (S.M.); kfobi@okstate.edu (K.F.); eametse@okstate.edu (E.A.)
* Correspondence: Correspondence: richard.a.bunce@okstate.edu; Tel.: +1-405-744-5952

Compound **page**

^{1}H and ^{13}C NMR for Methyl 1-Isobutyl-5-nitro-2-phenyl-1*H*-indole-3-carboxylate (**2**) ……….………….………….. 2

^{1}H and ^{13}C NMR for Methyl 1-Benzyl-2-methyl-5-nitro-1*H*-indole-3-carboxylate (**3**) ……..………….….……….. 3

^{1}H and ^{13}C NMR for Methyl 2-Methyl-1-(4-methylbenzyl)-5-nitro-1*H*-indole-3-carboxylate (**4**) ……….…....……..... 4

^{1}H and ^{13}C NMR for Methyl 1-(4-Chlorobenzyl)-1-methyl-5-nitro-1*H*-indole-3-carboxylate (**5**) …...…...……...…… 5

^{1}H and ^{13}C NMR for Methyl 1-(2-Chlorobenzyl)-2-methyl-5-nitro-1*H*-indole-3-carboxylate (**6**) ….……..…………… 6

^{1}H and ^{13}C NMR for Methyl 1-Benzyl-5-nitro-2-phenyl-1*H*-indole-3-carboxylate (**7**) ……………..…………..... 7

^{1}H and ^{13}C NMR for Methyl 5-Nitro-1-phenethyl-2-phenyl-1*H*-indole-3-carboxylate (**8**) …………..……..…………. 8

^{1}H and ^{13}C NMR for Methyl 1-(4-Methylbenzyl)-5-nitro-2-phenyl-1*H*-indole-3-carboxylate (**9**) ……….…….…… 9

^{1}H and ^{13}C NMR for Methyl 1-Benzyl-2-(4-fluorophenyl)-5-nitro-1*H*-indole-3-carboxylate (**10**) ……….……….... 10

^{19}F NMR for Methyl 1-Benzyl-2-(4-fluorophenyl)-5-nitro-1*H*-indole-3-carboxylate (**10**) …………….……….…….11

^{1}H and ^{13}C NMR for Methyl 1-Benzyl-2-(4-chlorophenyl)-5-nitro-1*H*-indole-3-carboxylate (**11**) ………..…...…. 12

^{1}H and ^{13}C NMR for Methyl 1-Benzyl-2-(2-chlorophenyl)-5-nitro-1*H*-indole-3-carboxylate (**12**) …………..……. 13

^{1}H and ^{13}C NMR for Methyl 1-Benzyl-2-(3-methoxyphenyl)-5-nitro-1*H*-indole-3-carboxylate (**13**) ..……..……. 14

^{1}H and ^{13}C NMR for Methyl 2-(4-Methylphenyl)-5-nitro-1-phenethyl-1*H*-indole-3-carboxylate (**14**) ……..……. 15

^{1}H and ^{13}C NMR for Methyl 2-(4-Fluorophenyl)-5-nitro-1-phenethyl-1*H*-indole-3-carboxylate (**15**) …….…..…. 16

^{19}F NMR for Methyl 2-(4-fluorophenyl)-5-nitro-1-phenethyl-1*H*-indole-3-carboxylate (**15**) ……………….…..…. 17

^{1}H and ^{13}C NMR for Methyl 2-(4-Methoxyphenyl)-5-nitro-1-phenethyl-1*H*-indole-3-carboxylate (**16**) …..…..….. 18

^{1}H and ^{13}C NMR for Methyl 1-Benzyl-5-cyano-2-phenyl-1*H*-indole-3-carboxylate (**17**) ……………….….….…… 19

^{1}H and ^{13}C NMR for Methyl 5-Cyano-1-phenethyl-2-phenyl-1*H*-indole-3-carboxylate (**18**) ……….…………..…. 20

^{1}H and ^{13}C NMR for Methyl 5-Cyano-2-(4-methylphenyl)-1-phenethyl-1*H*-indole-3-carboxylate (**19**) ……..….… 21

^{1}H and ^{13}C NMR for Methyl 5-Cyano-2-(4-fluorophenyl)-1-phenethyl-1*H*-indole-3-carboxylate (**20**) ……......… 22

^{19}F NMR for: Methyl 5-Cyano-2-(4-fluorophenyl)-1-phenethyl-1*H*-indole-3-carboxylate (**20**) ……….…..….….. 23

^{1}H and ^{13}C NMR for Methyl 2-(4-Chlorophenyl)-5-cyano-1-phenethyl-1*H*-indole-3-carboxylate (**21**) ……...…… 24

^{1}H and ^{13}C NMR for Methyl 5-Cyano-2-(4-methoxyphenyl)-1-phenethyl-1*H*-indole-3-carboxylate (**22**) …...…… 25

^{1}H and ^{13}C NMR for Methyl 1-Isobutyl-5-nitro-2-phenyl-1*H*-indole-3-carboxylate (**2**)

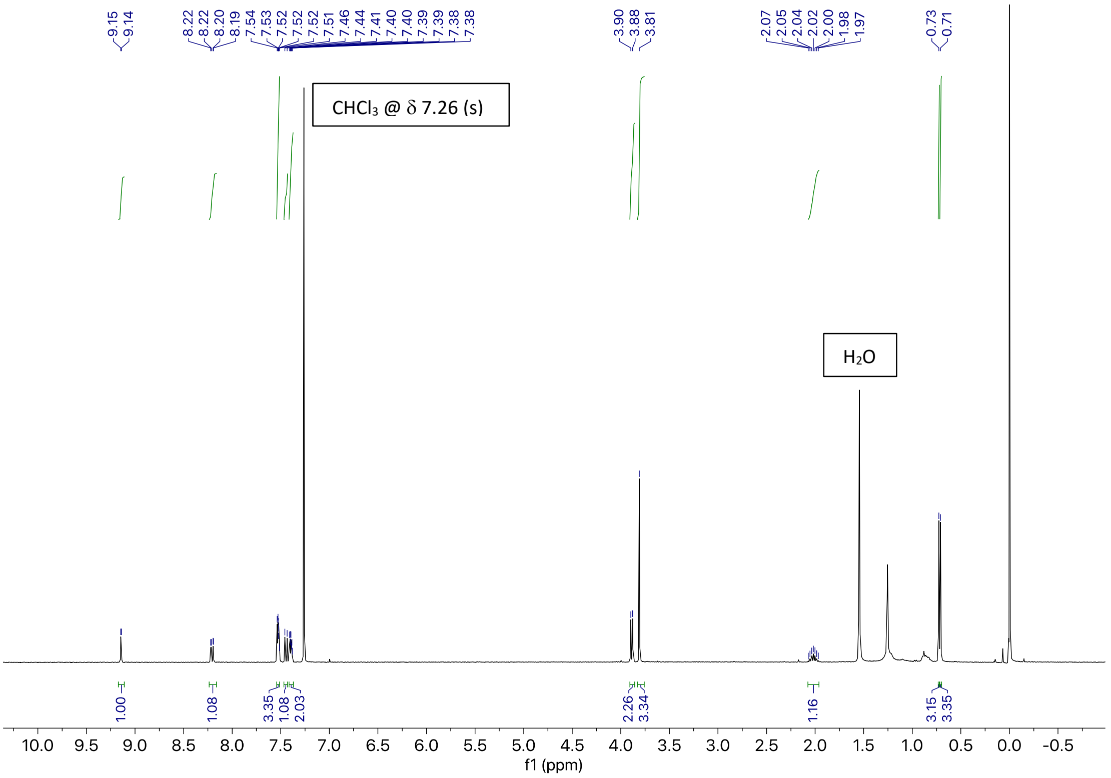

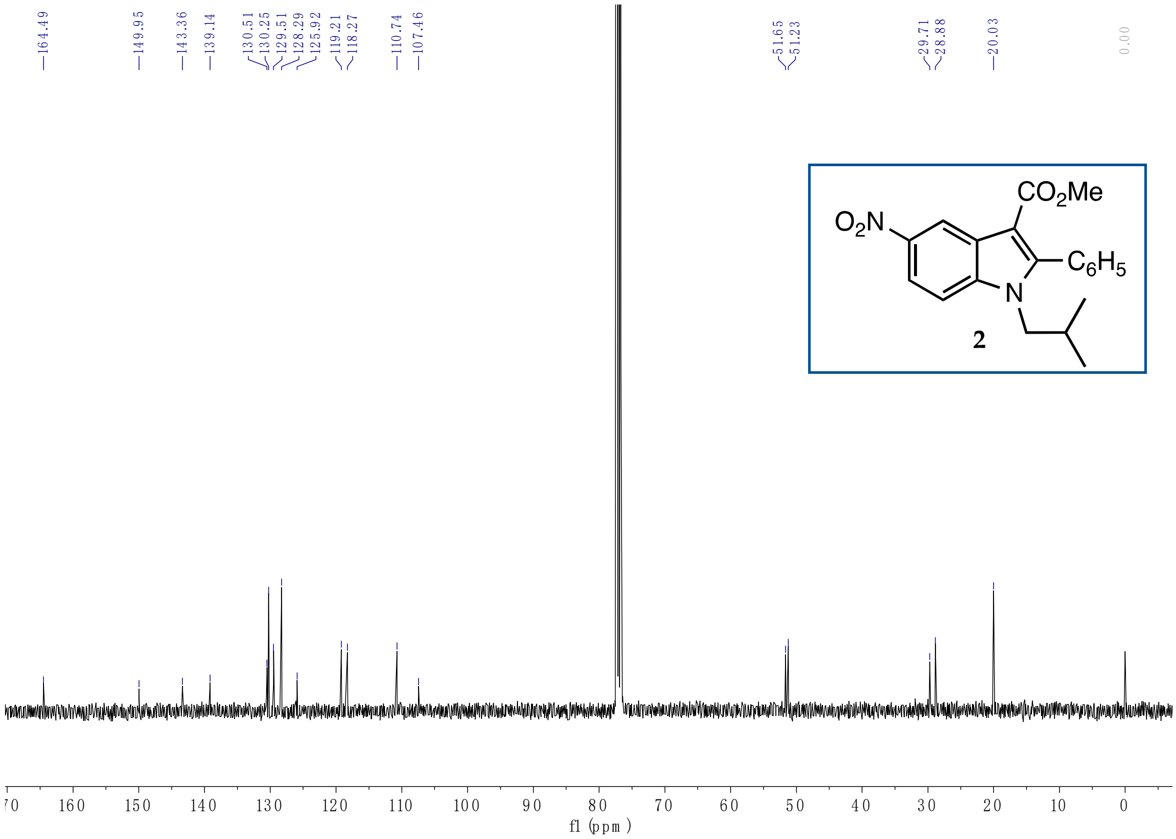

^{1}H and ^{13}C NMR for Methyl 1-Benzyl-2-methyl-5-nitro-1*H*-indole-3-carboxylate (**3**)

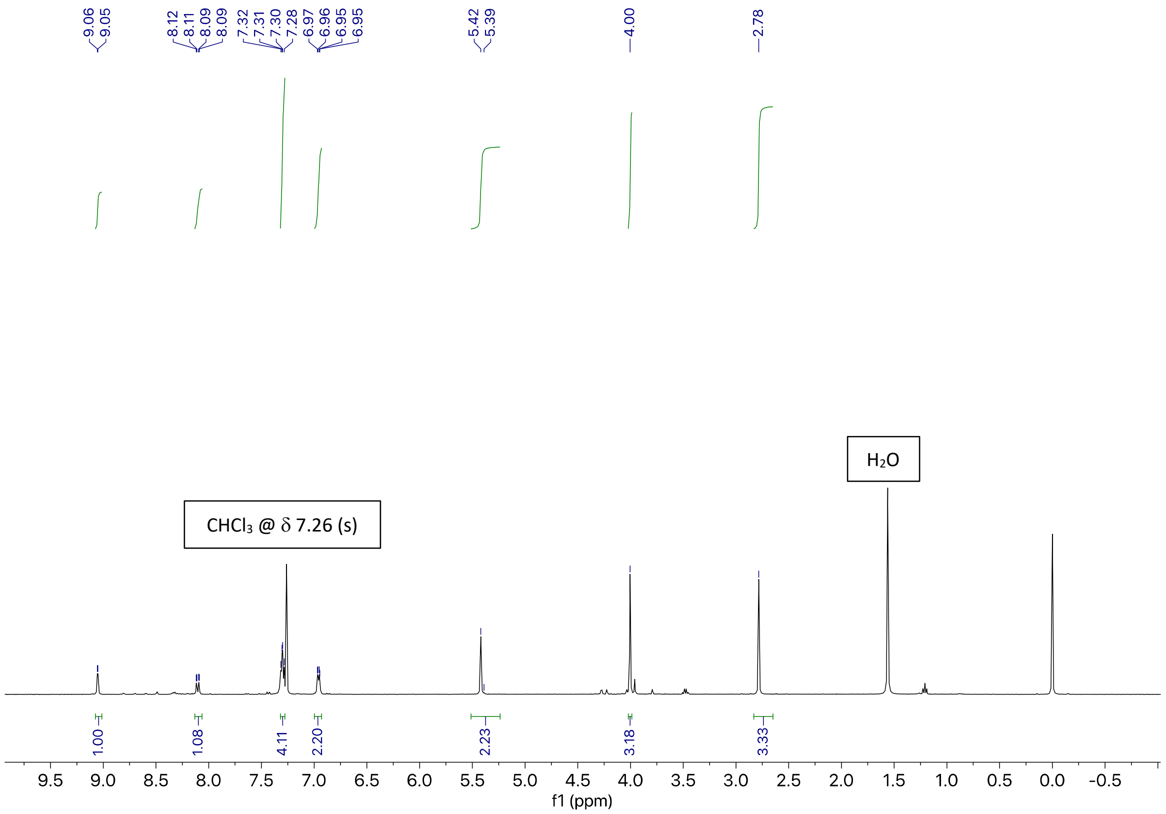
9.06
9.05
8.12
8.11
8.09
8.09
7.32
7.31
7.30
7.28
6.97
6.96
6.95
6.95
5.42
5.39
4.00
2.78
H₂O
CHCl₃ @ δ 7.26 (s)
1.00
1.08
4.11
2.20
2.23
3.18
3.33
9.5 9.0 8.5 8.0 7.5 7.0 6.5 6.0 5.5 5.0 4.5 4.0 3.5 3.0 2.5 2.0 1.5 1.0 0.5 0.0 -0.5
f1 (ppm)

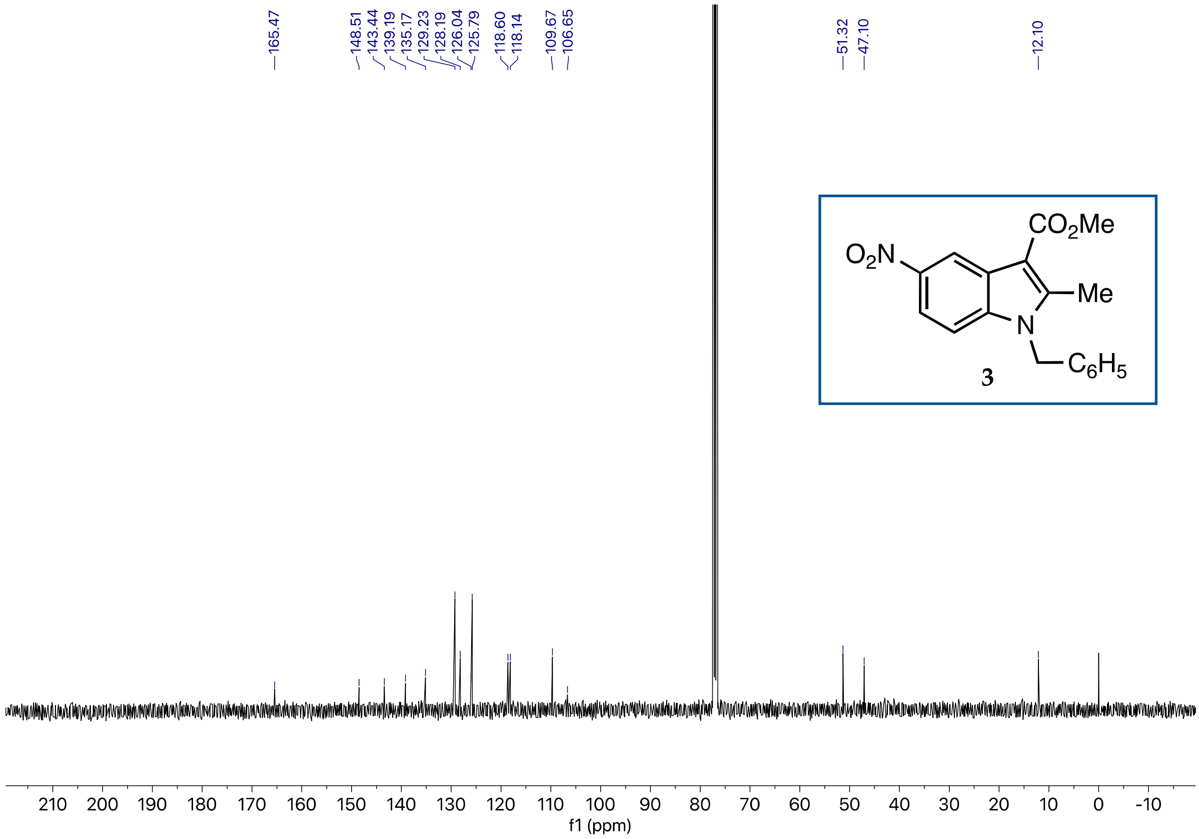
165.47
148.51
143.44
139.19
135.17
129.23
128.19
126.04
125.79
118.60
118.14
109.67
106.65
51.32
47.10
12.10
210 200 190 180 170 160 150 140 130 120 110 100 90 80 70 60 50 40 30 20 10 0 -10
f1 (ppm)
CO₂Me
O₂N
Me
N
C₆H₅

3

^{1}H and ^{13}C NMR for Methyl 2-Methyl-1-(4-methylbenzyl)-5-nitro-1*H*-indole-3-carboxylate (**4**)

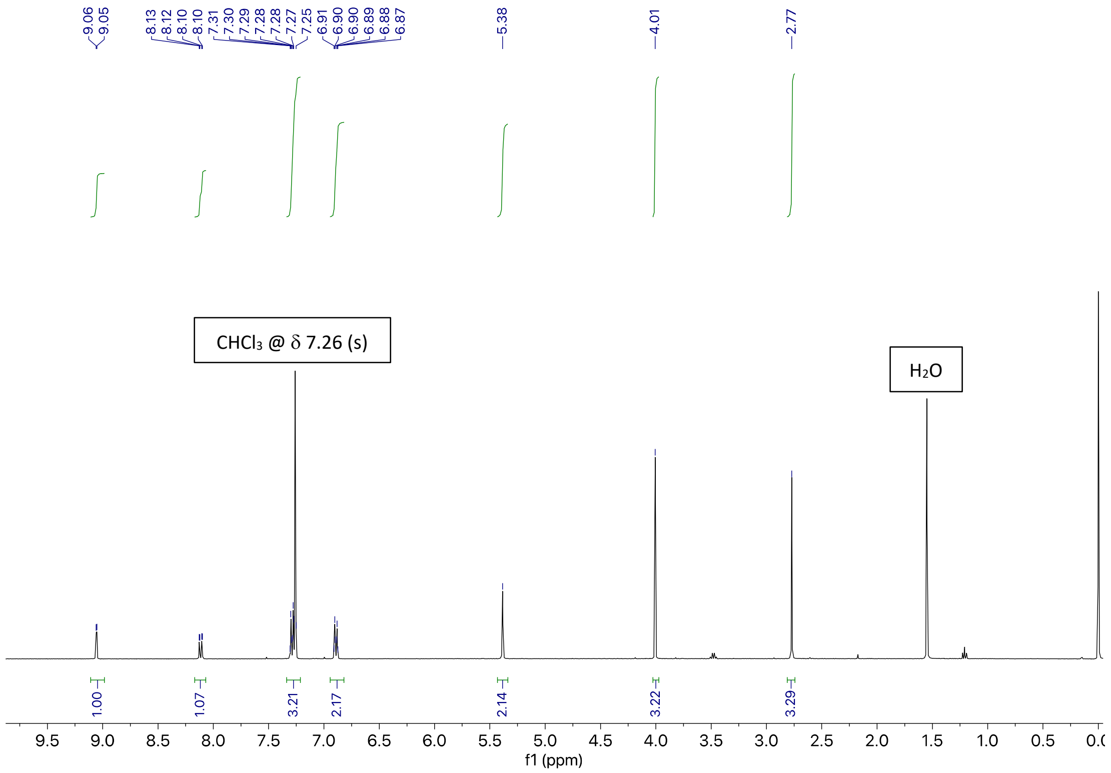

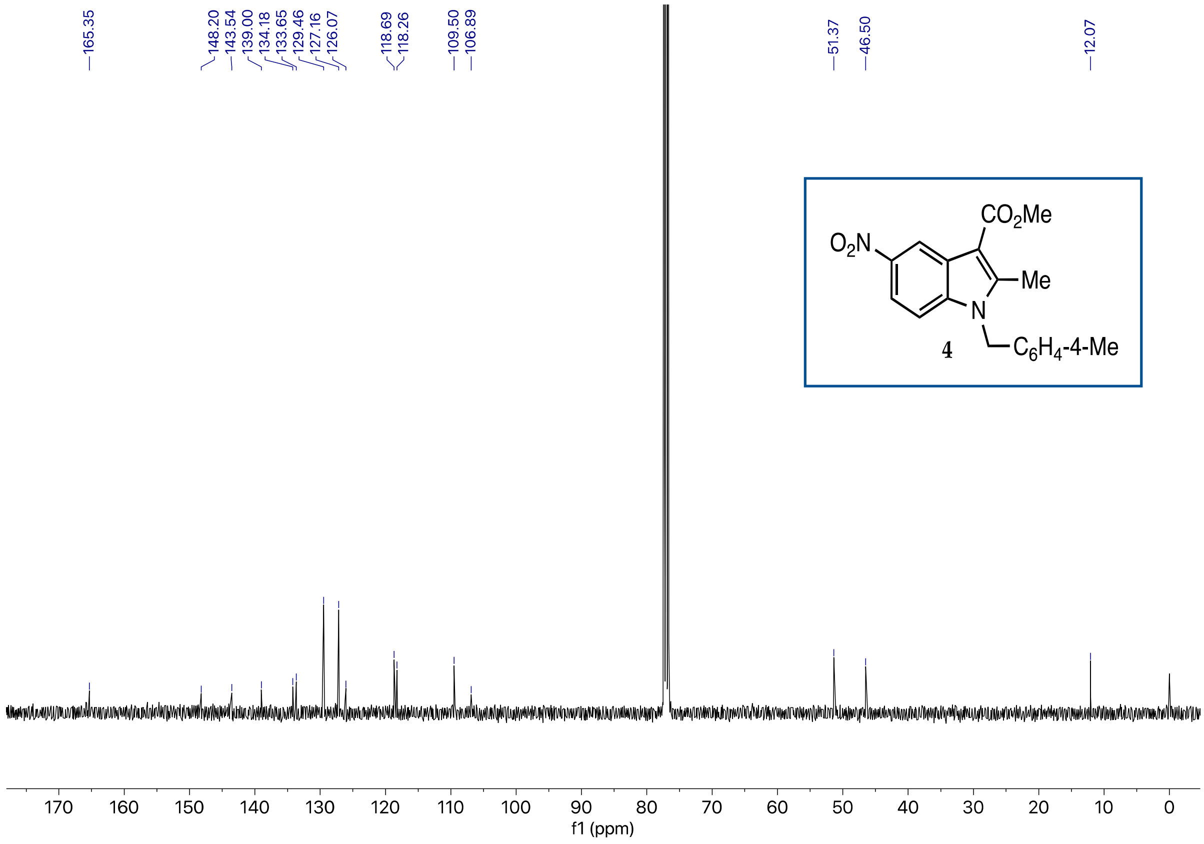

^{1}H and ^{13}C NMR for Methyl 1-(4-Chlorobenzyl)-1-methyl-5-nitro-1*H*-indole-3-carboxylate (**5**)

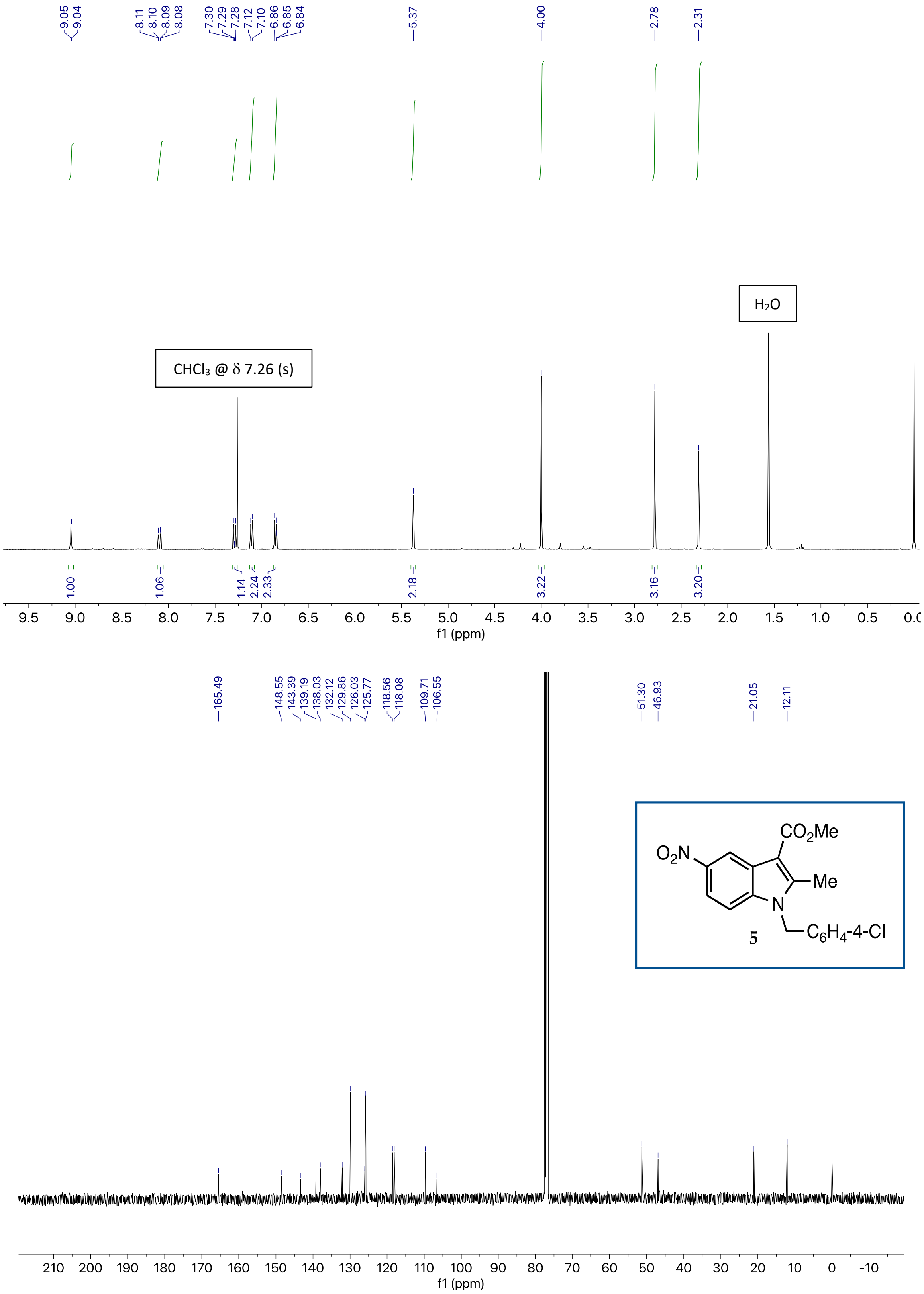

^{1}H and ^{13}C NMR for Methyl 1-(2-Chlorobenzyl)-2-methyl-5-nitro-1*H*-indole-3-carboxylate (**6**)

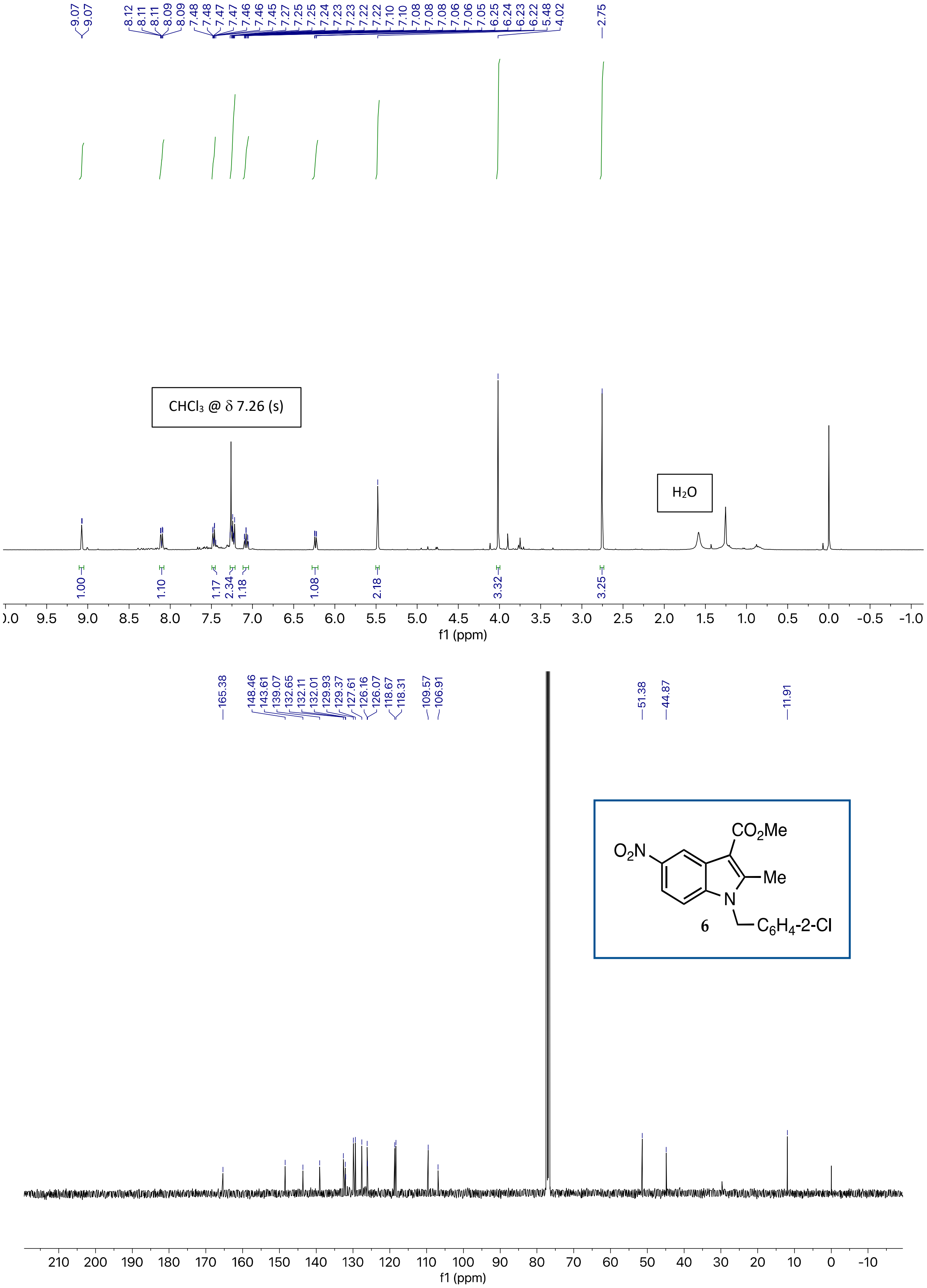

^{1}H and ^{13}C NMR for Methyl 1-Benzyl-5-nitro-2-phenyl-1*H*-indole-3-carboxylate (7)

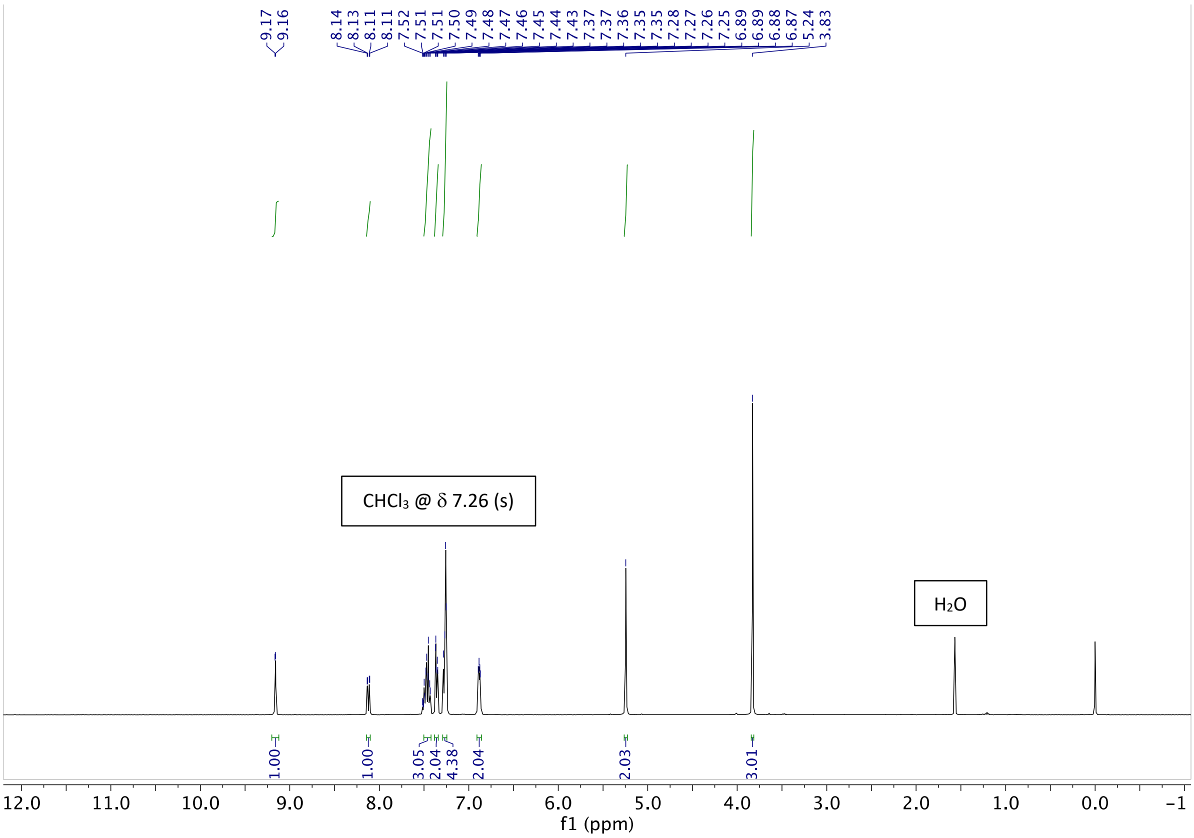
9.17 9.16 8.14 8.13 8.11 8.11 7.52 7.51 7.51 7.50 7.49 7.48 7.47 7.46 7.45 7.44 7.43 7.37 7.36 7.35 7.35 7.28 7.27 7.26 7.25 6.89 6.88 6.87 5.24 3.83
CHCl3 @ δ 7.26 (s)
H2O
1.00 1.00 3.05 2.04 4.38 2.04 2.03 3.01
12.0 11.0 10.0 9.0 8.0 7.0 6.0 5.0 4.0 3.0 2.0 1.0 0.0 -1
f1 (ppm)

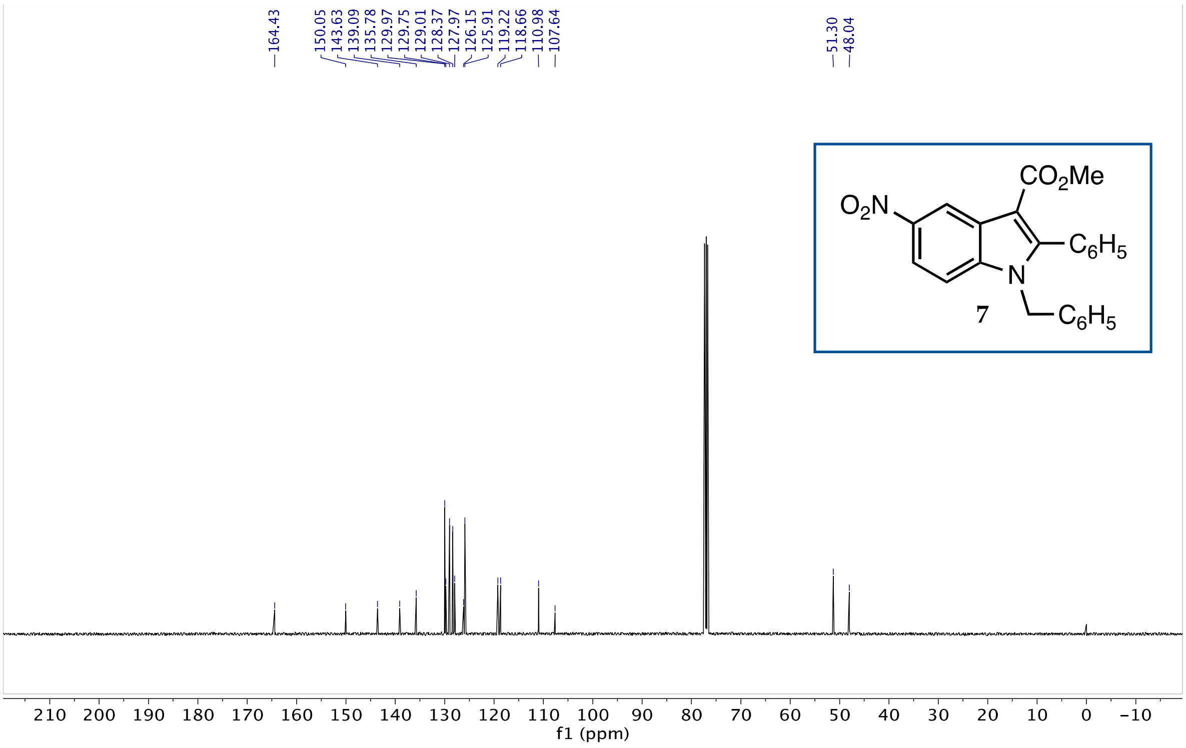
164.43
150.05 143.63 139.09 135.78 129.97 129.75 129.01 128.37 127.97 126.15 125.91 119.22 118.66 110.98 107.64
51.30 48.04
210 200 190 180 170 160 150 140 130 120 110 100 90 80 70 60 50 40 30 20 10 0 -10
f1 (ppm)
CO2Me
O2N
C6H5
N
C6H5

7

^{1}H and ^{13}C NMR for Methyl 5-Nitro-1-phenethyl-2-phenyl-1*H*-indole-3-carboxylate (**8**)

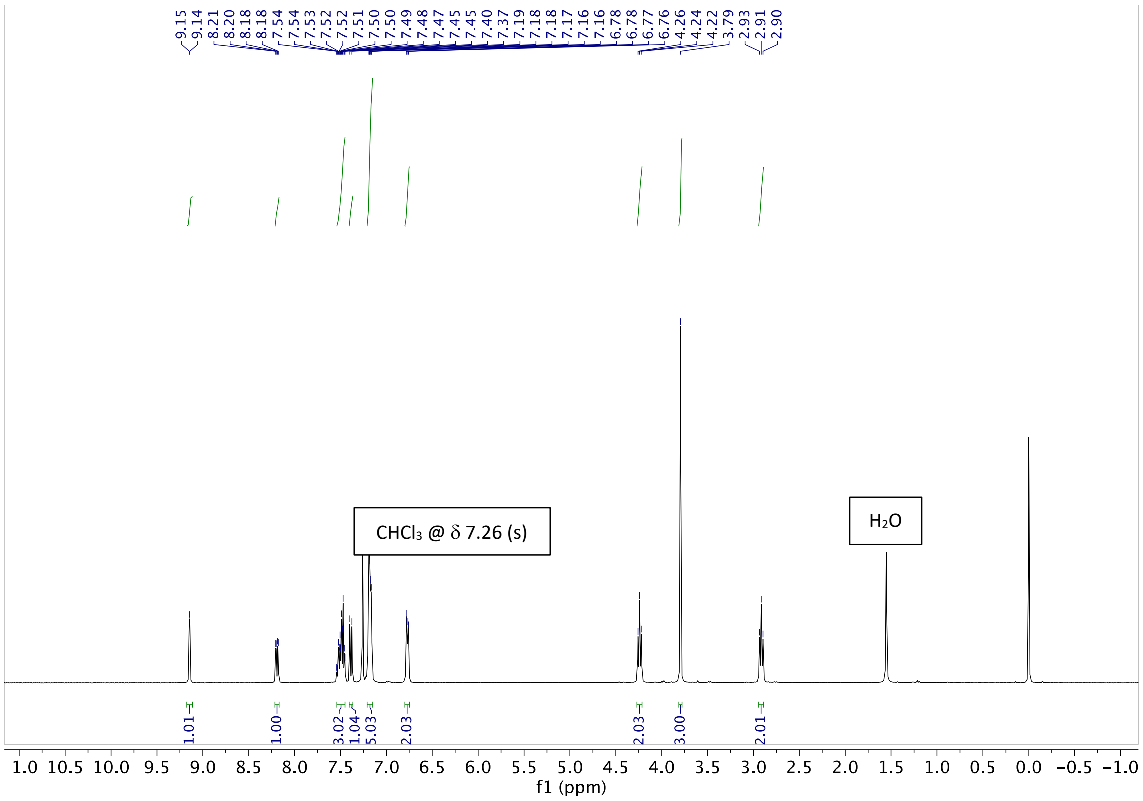

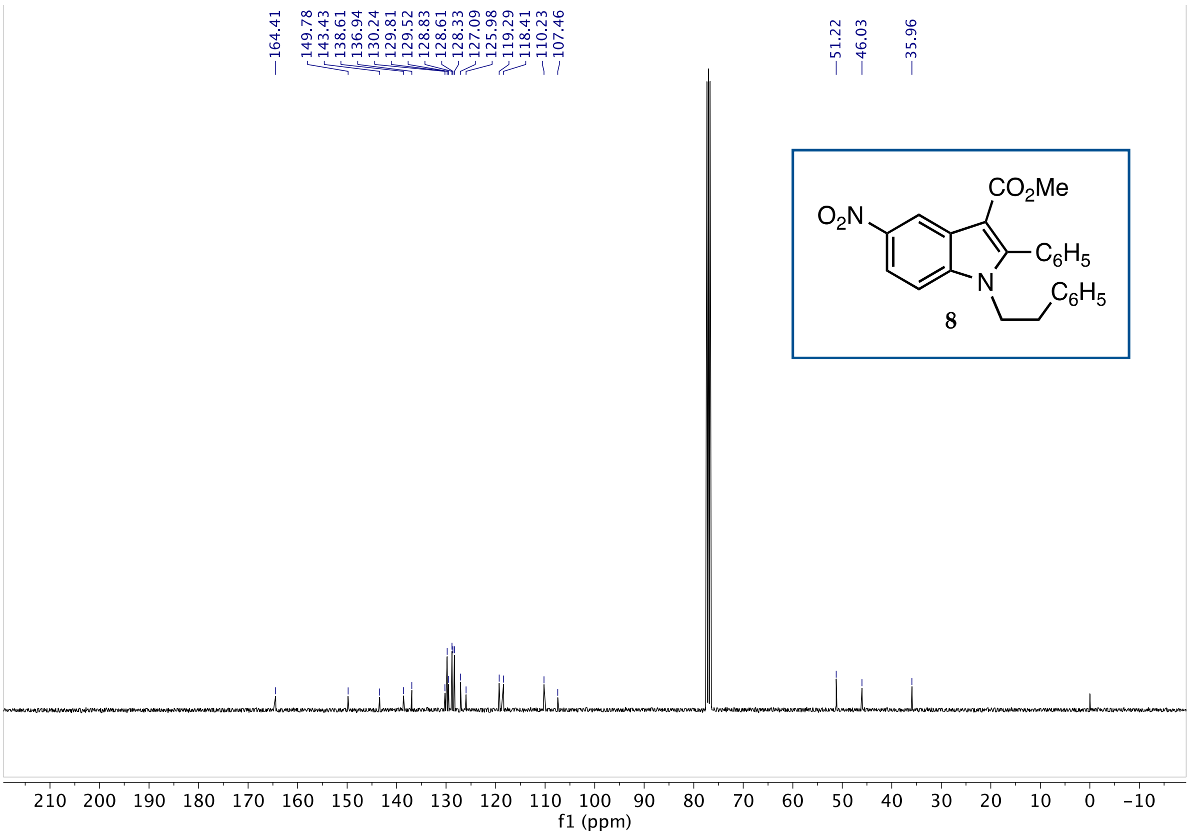

^{1}H and ^{13}C NMR for Methyl 1-(4-Methylbenzyl)-5-nitro-2-phenyl-1*H*-indole-3-carboxylate (**9**)

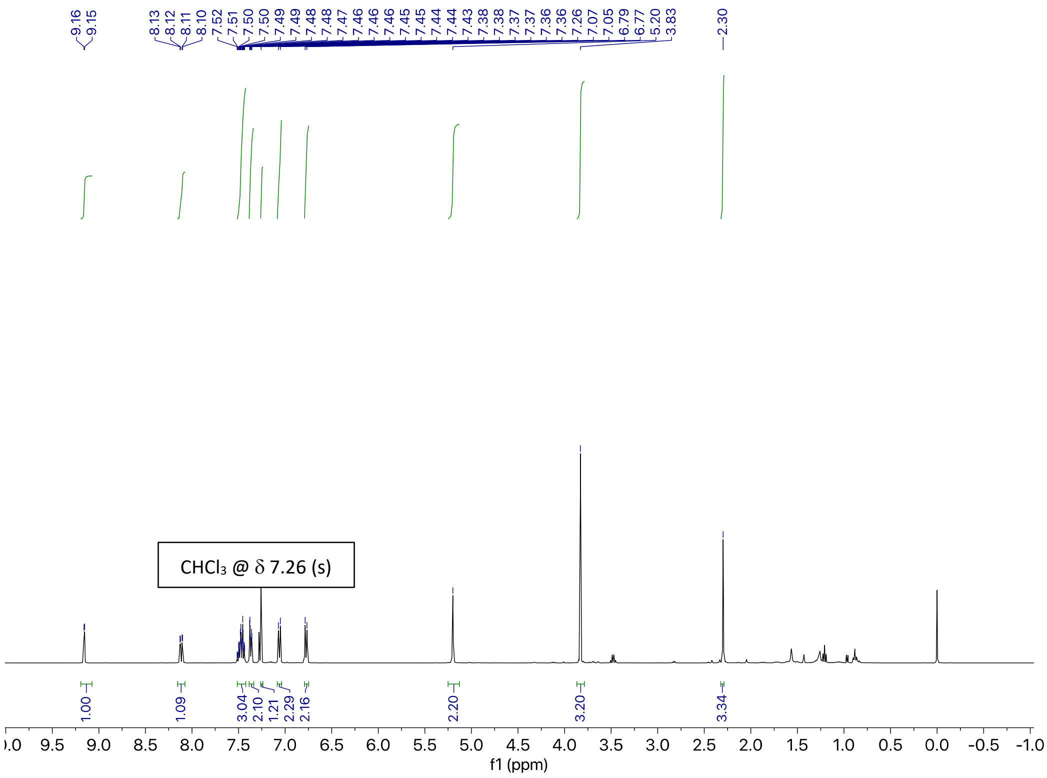

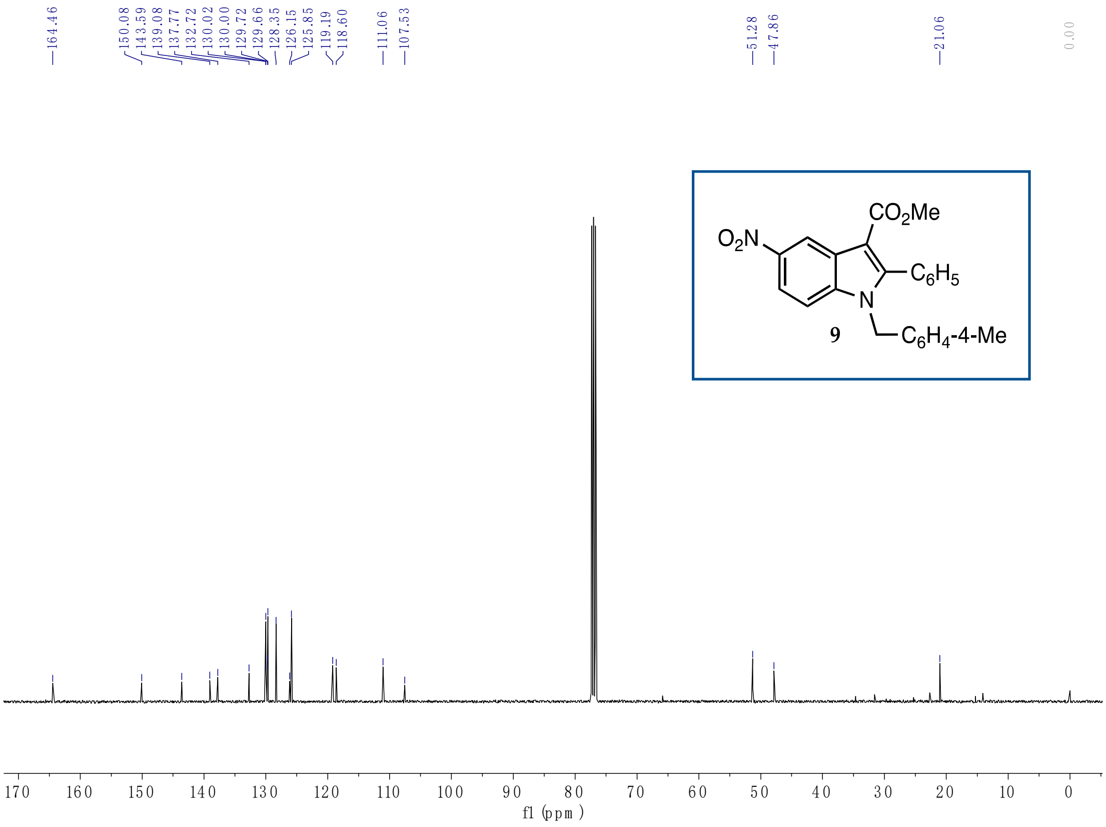

^{1}H and ^{13}C NMR for Methyl 1-Benzyl-2-(4-fluorophenyl)-5-nitro-1*H*-indole-3-carboxylate (**10**)

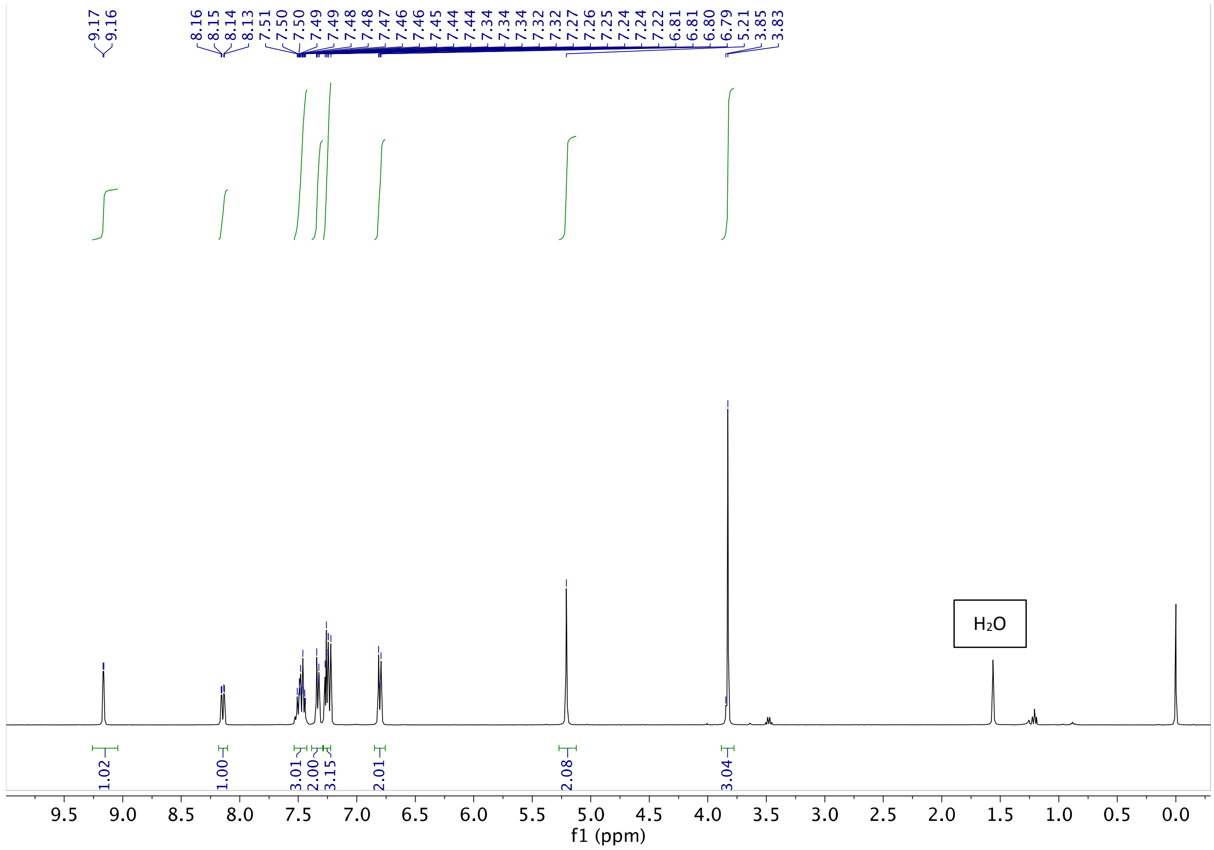

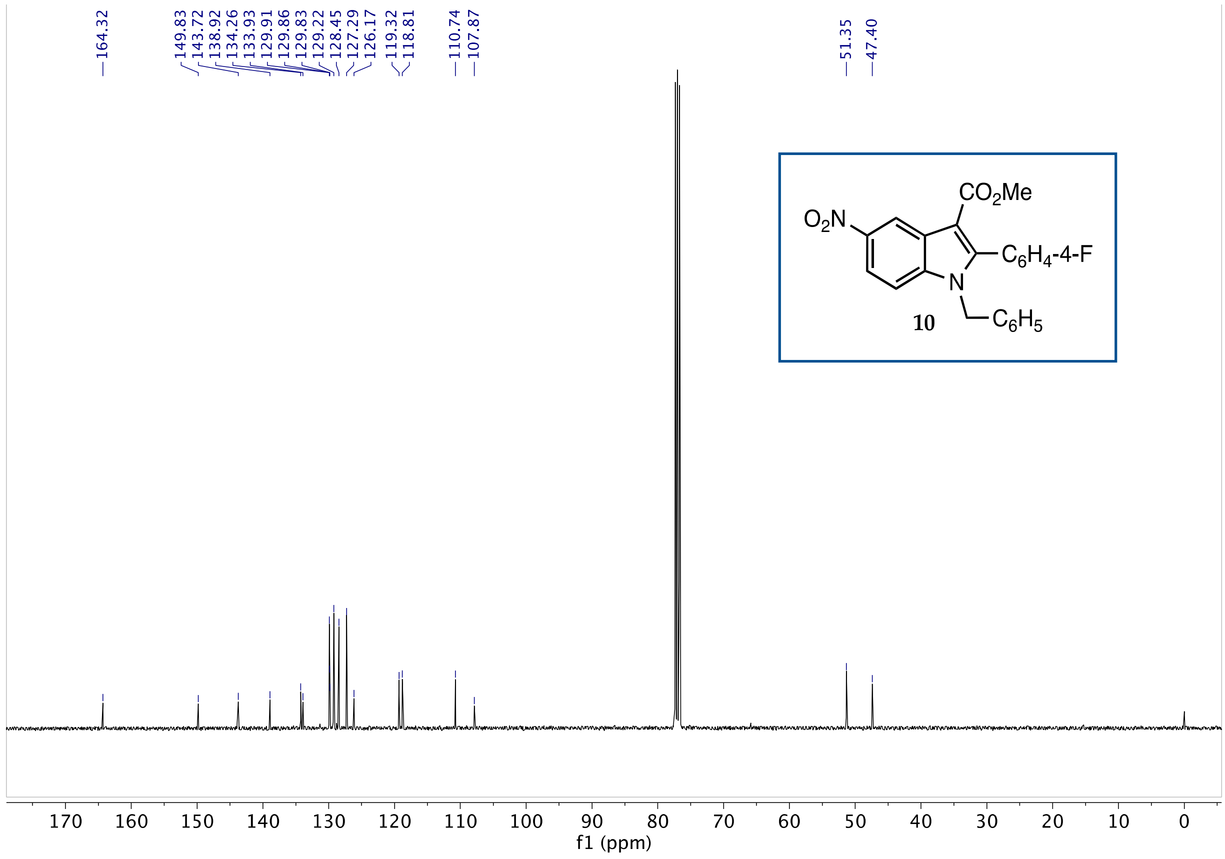

^{19}F NMR for Methyl 1-Benzyl-2-(4-fluorophenyl)-5-nitro-1H-indole-3-carboxylate (**10**)

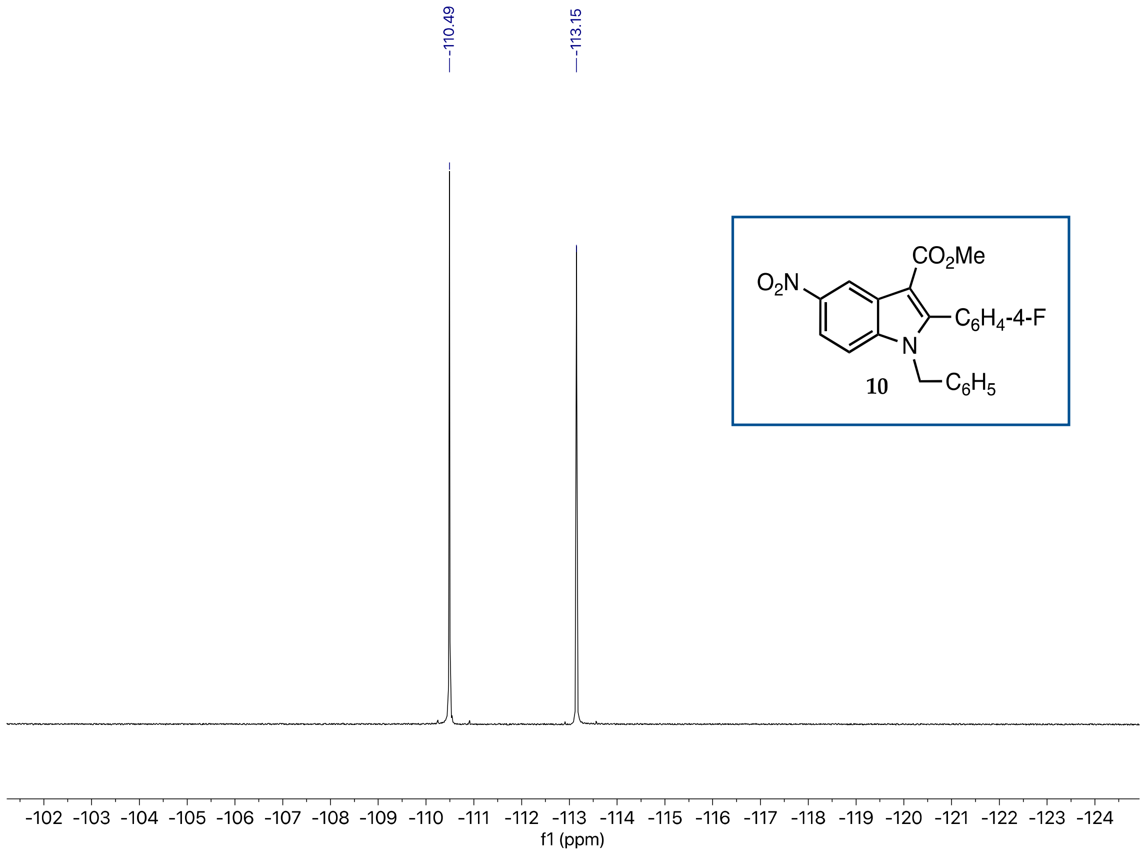
-110.49
-113.15
-102 -103 -104 -105 -106 -107 -108 -109 -110 -111 -112 -113 -114 -115 -116 -117 -118 -119 -120 -121 -122 -123 -124
f1 (ppm)
CO2Me
O2N
C6H4-4-F
N
C6H5
10

^{1}H and ^{13}C NMR for Methyl 1-Benzyl-2-(4-chlorophenyl)-5-nitro-1*H*-indole-3-carboxylate (**11**)

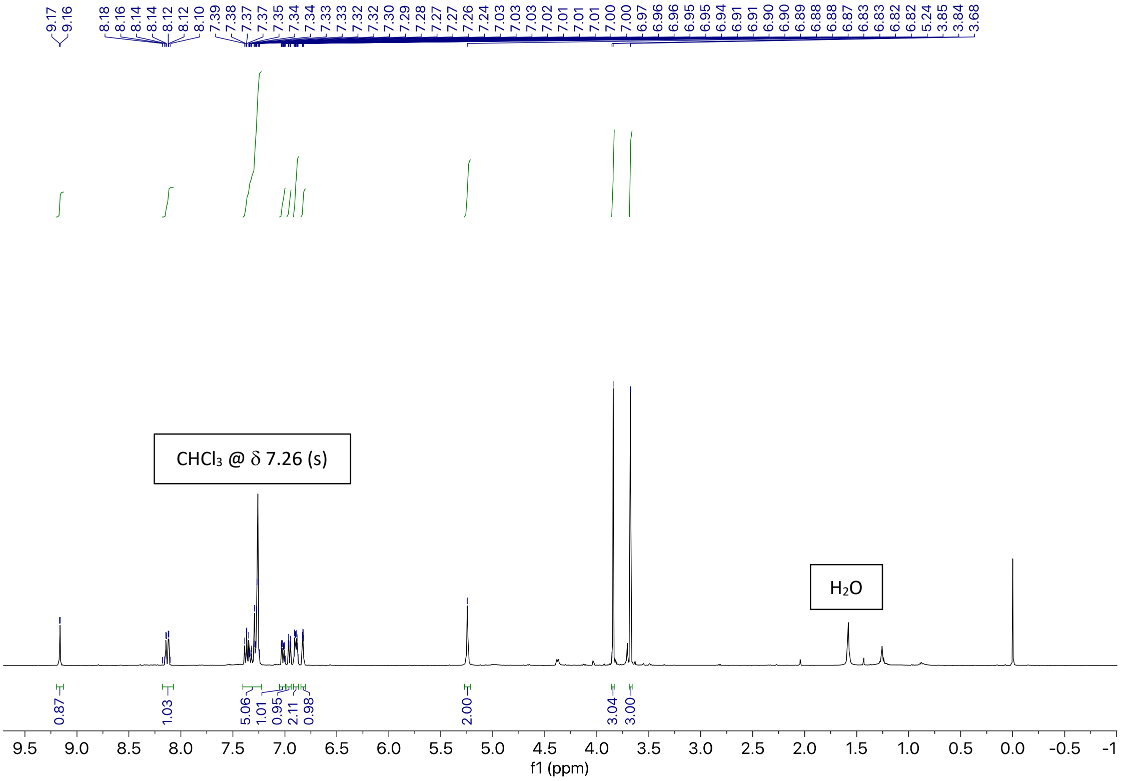

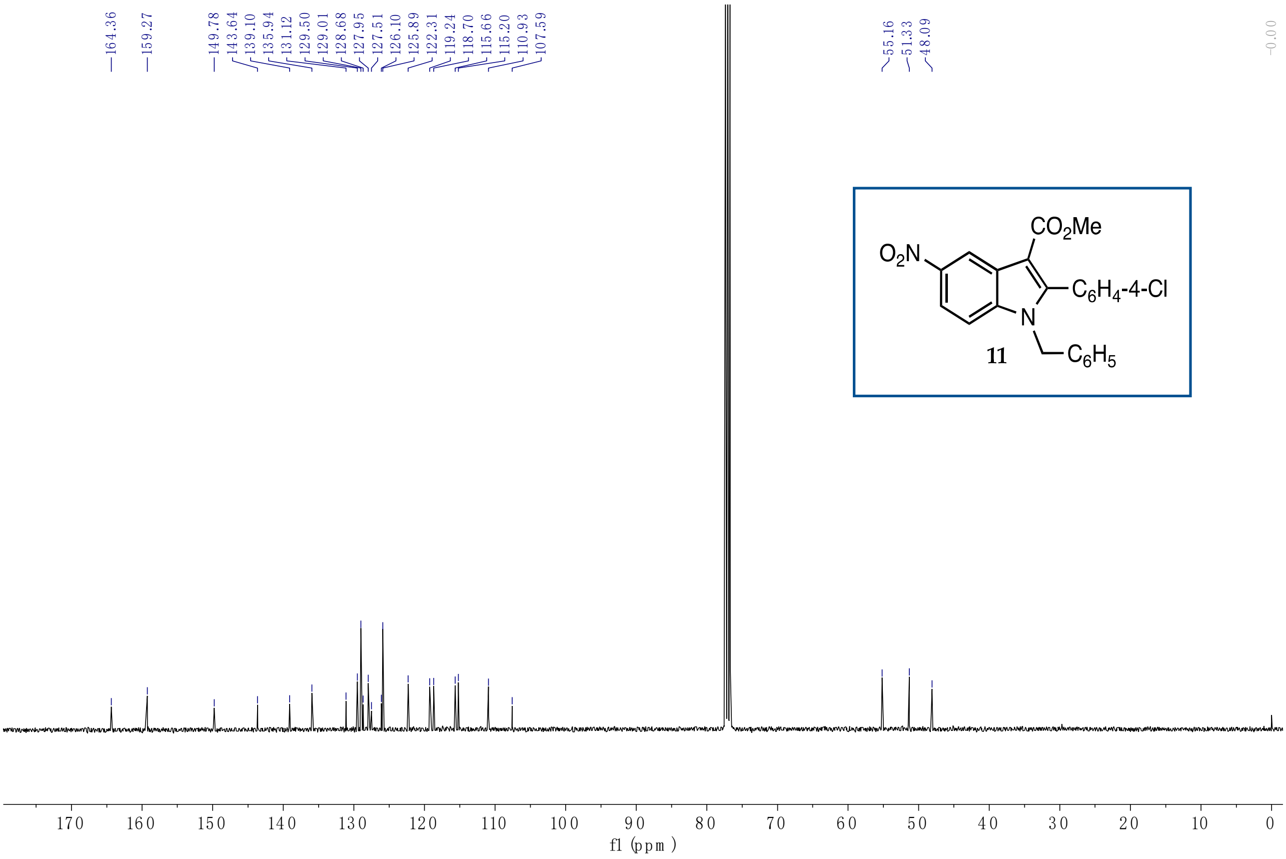

^{1}H and ^{13}C NMR for Methyl 1-Benzyl-2-(2-chlorophenyl)-5-nitro-1*H*-indole-3-carboxylate (**12**)

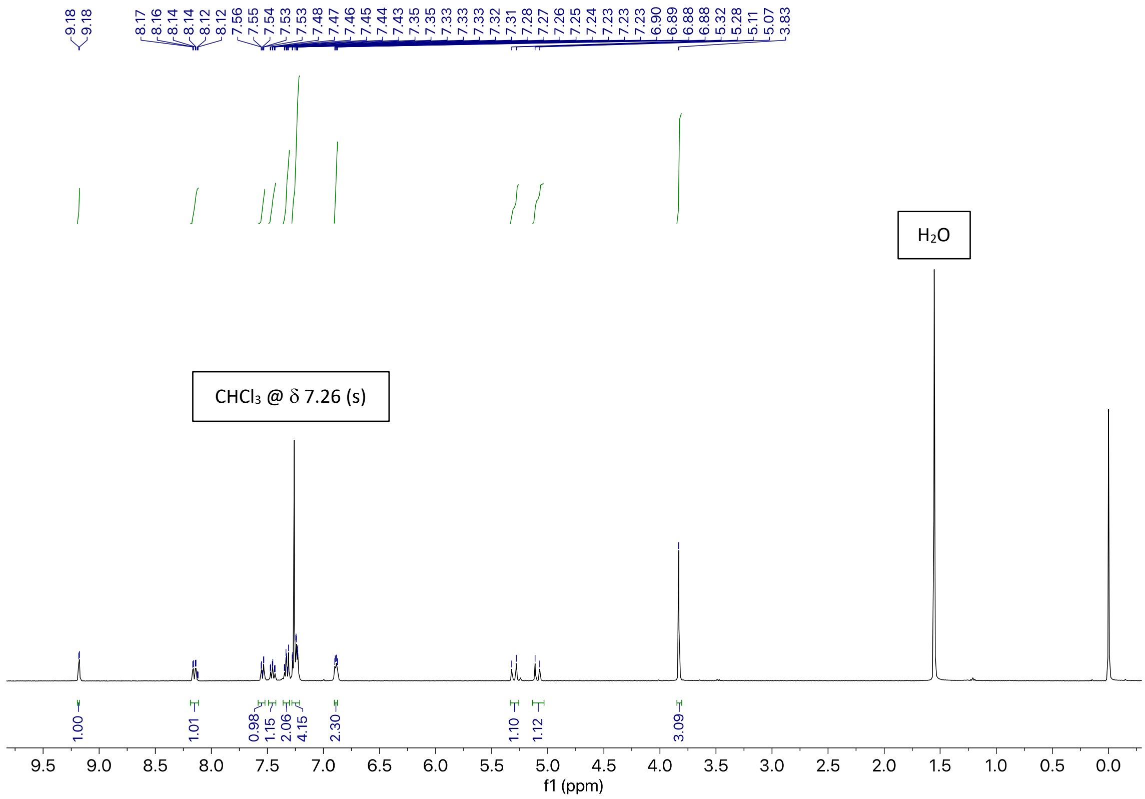

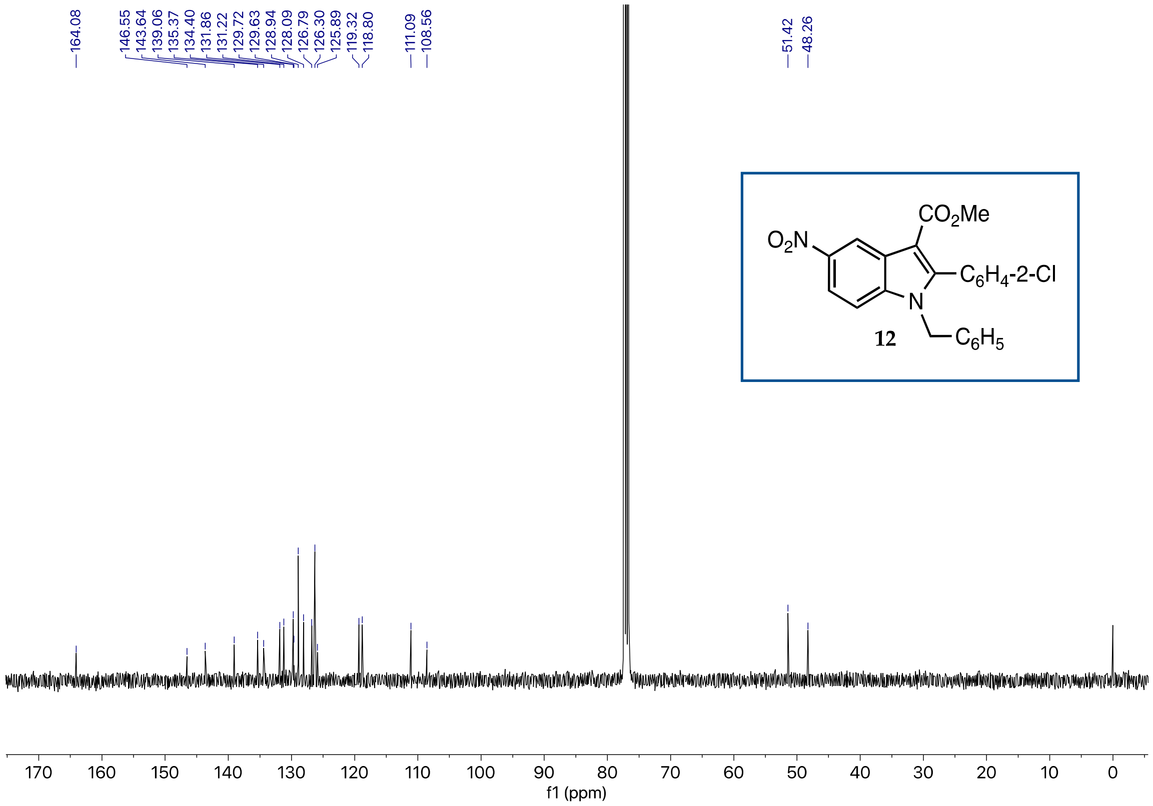

^{1}H and ^{13}C NMR for Methyl 1-Benzyl-2-(3-methoxyphenyl)-5-nitro-1*H*-indole-3-carboxylate (**13**)

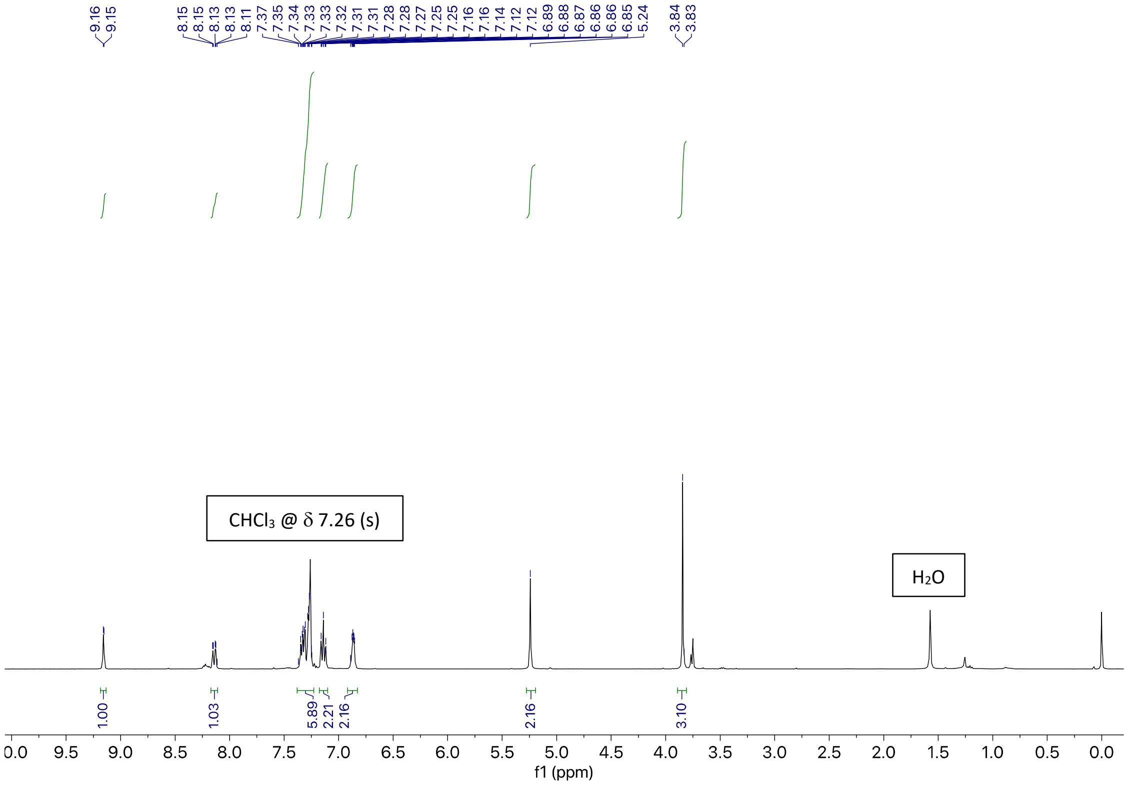

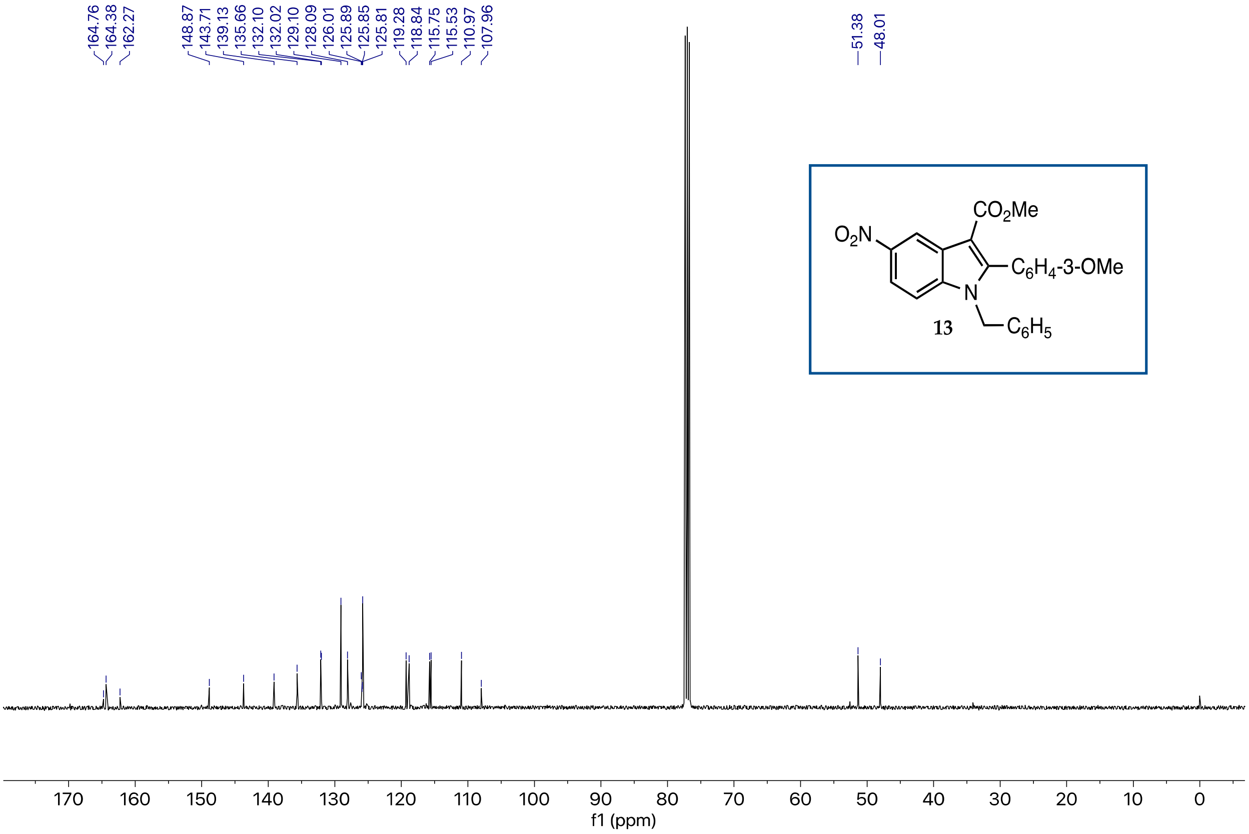

^{1}H and ^{13}C NMR for Methyl 2-(4-Methylphenyl)-5-nitro-1-phenethyl-1*H*-indole-3-carboxylate (**14**)

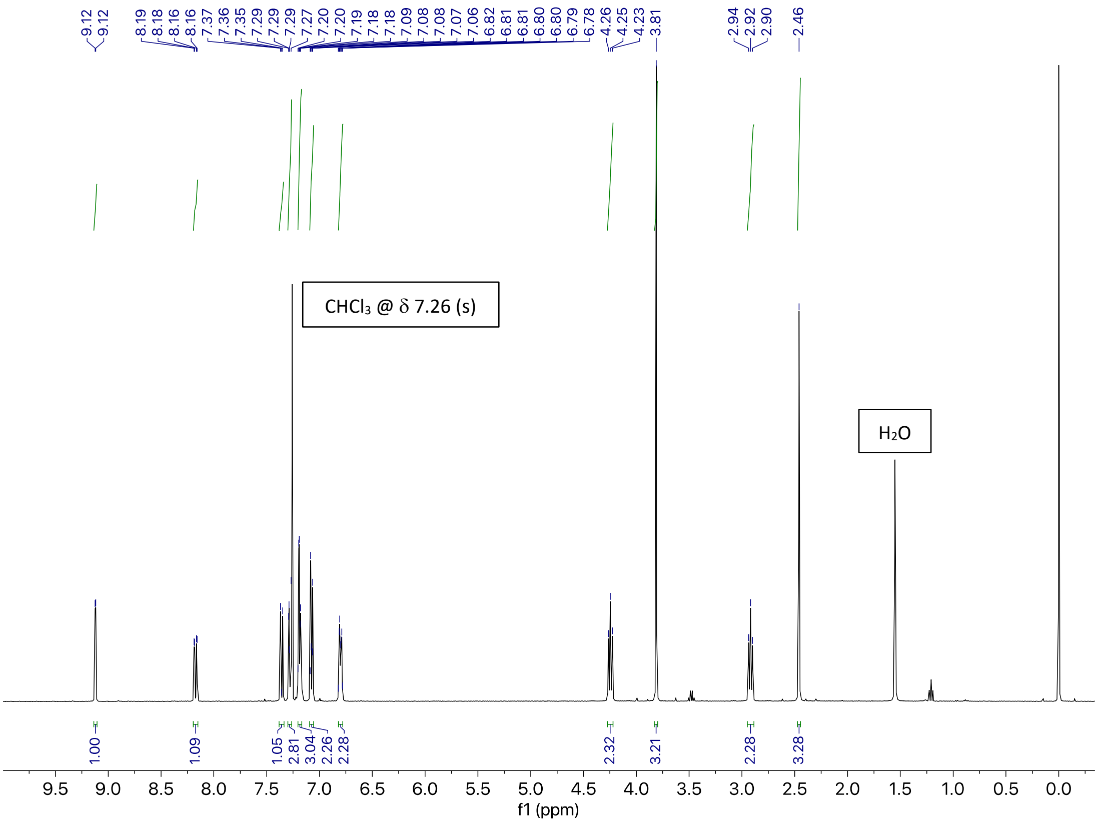

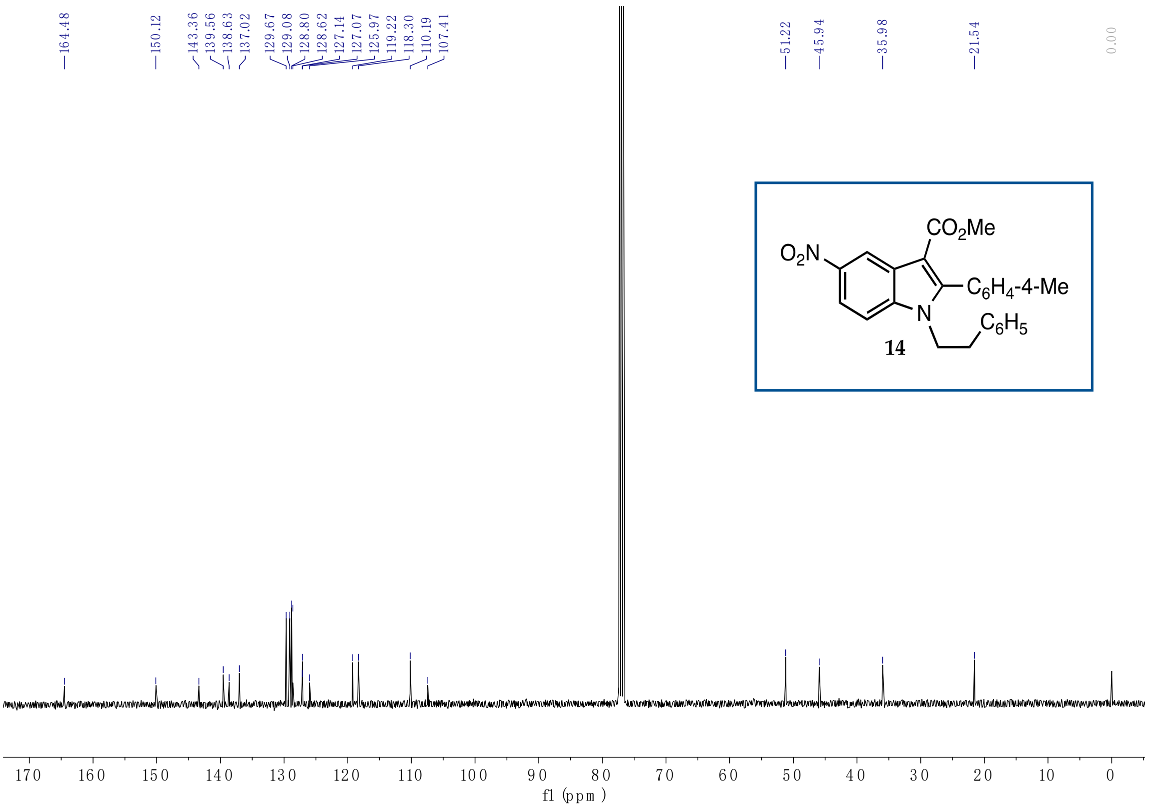

^{1}H and ^{13}C NMR for Methyl 2-(4-Fluorophenyl)-5-nitro-1-phenethyl-1*H*-indole-3-carboxylate (**15**)

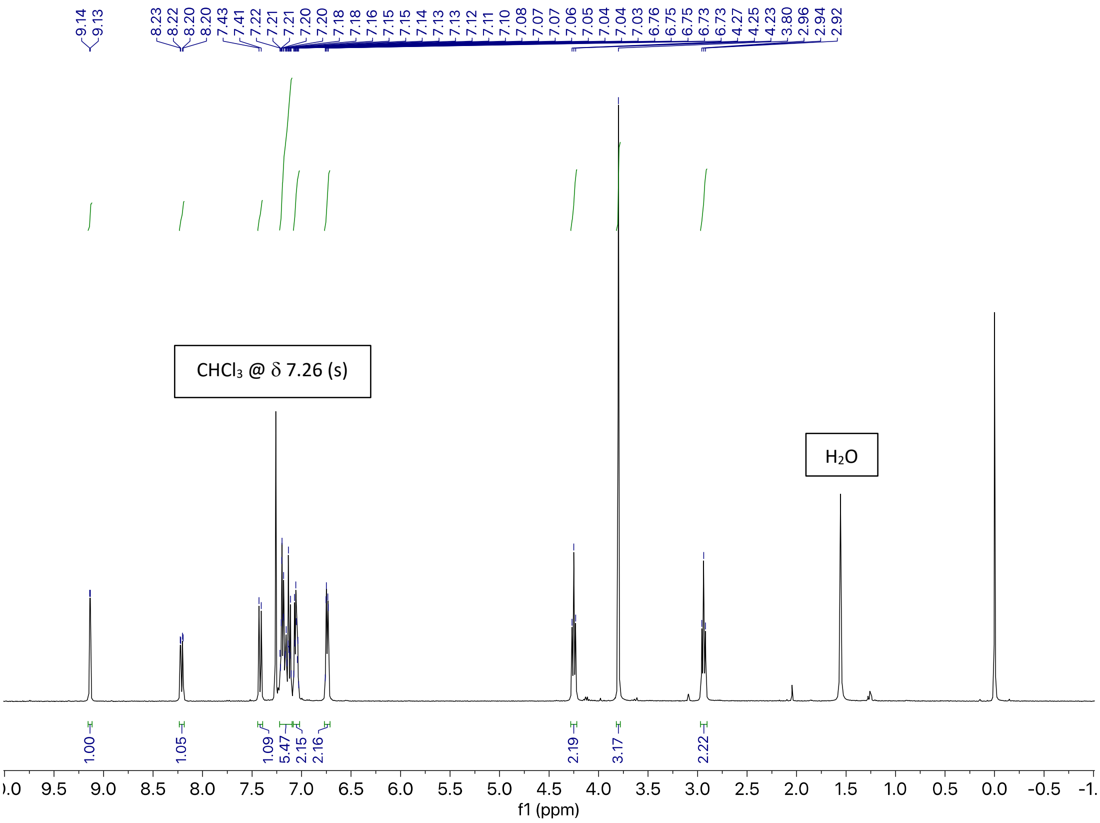

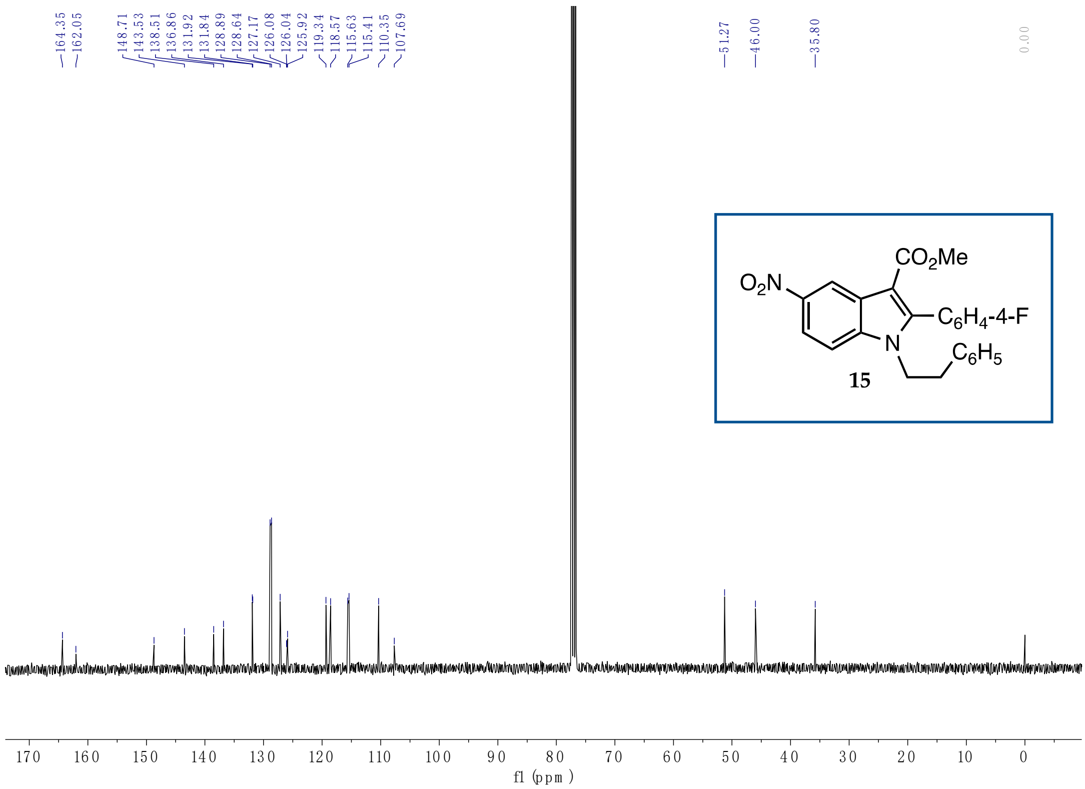

^{19}F NMR for Methyl 2-(4-Fluorophenyl)-5-nitro-1-phenethyl-1*H*-indole-3-carboxylate (**15**)

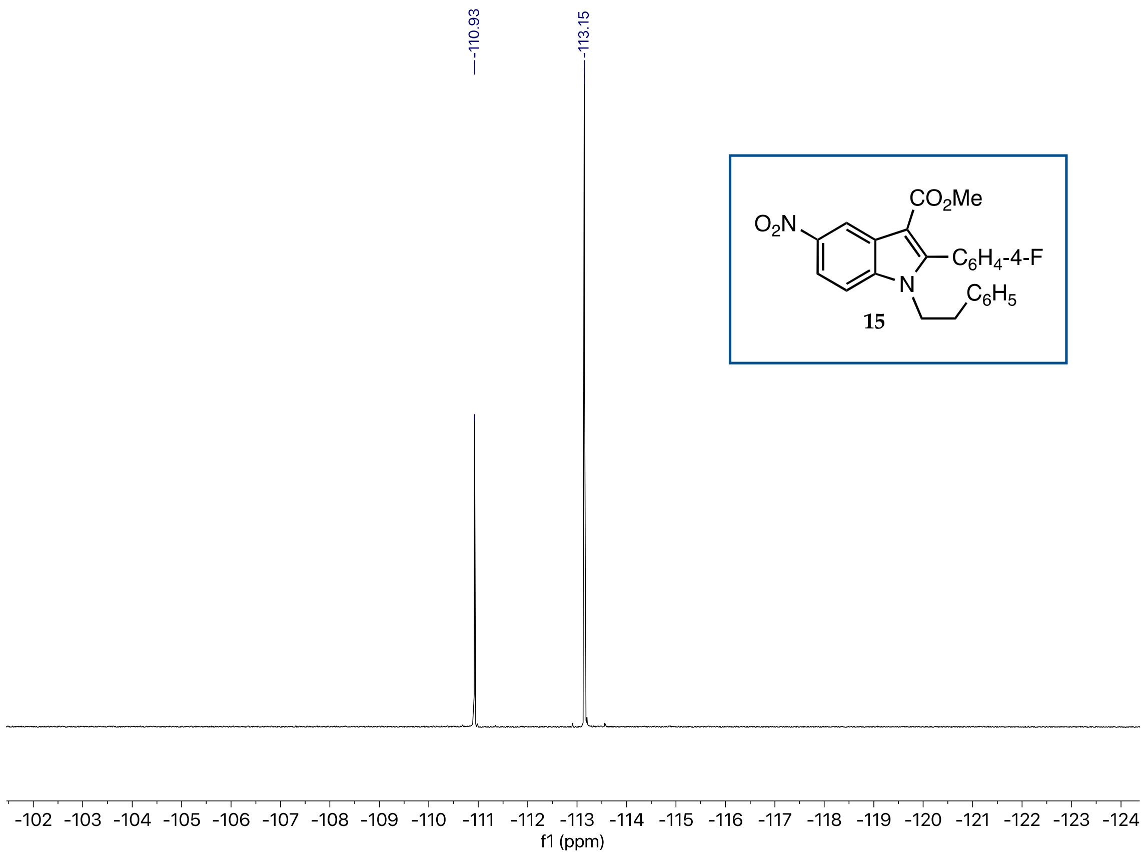

-110.93
-113.15
-102 -103 -104 -105 -106 -107 -108 -109 -110 -111 -112 -113 -114 -115 -116 -117 -118 -119 -120 -121 -122 -123 -124
f1 (ppm)
CO2Me
O2N
C6H4-4-F
N
C6H5
15

^{1}H and ^{13}C NMR for Methyl 2-(4-Methoxyphenyl)-5-nitro-1-phenethyl-1*H*-indole-3-carboxylate (**16**)

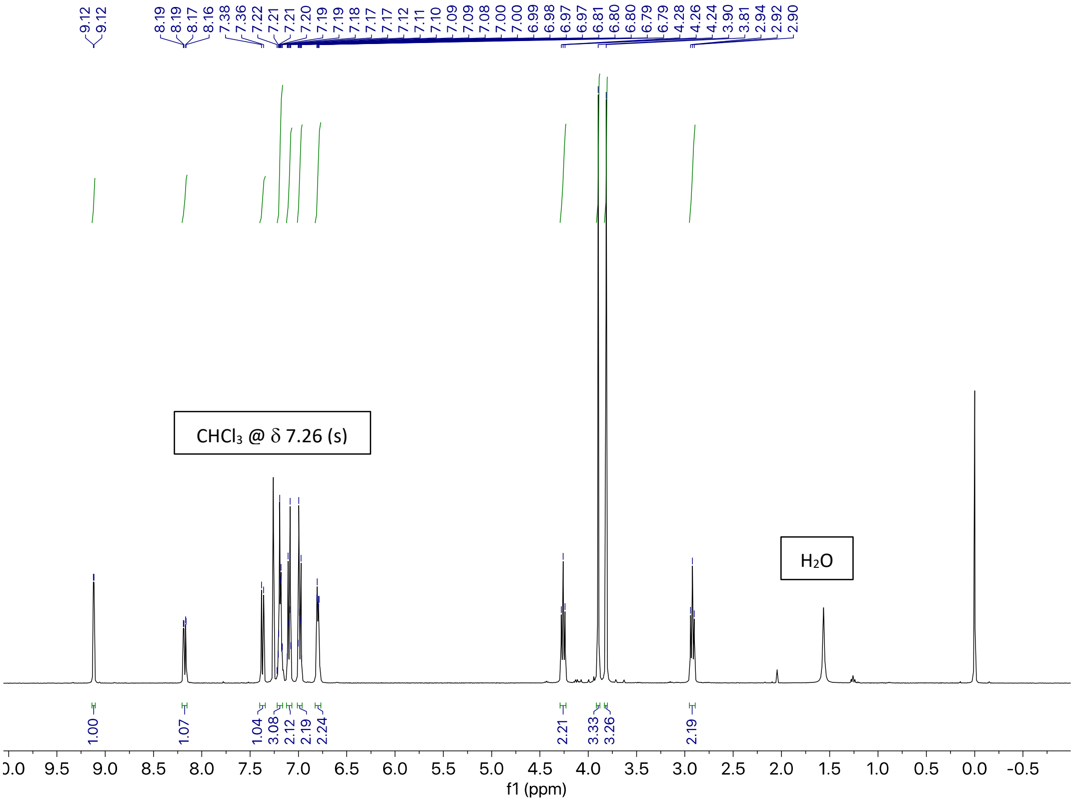

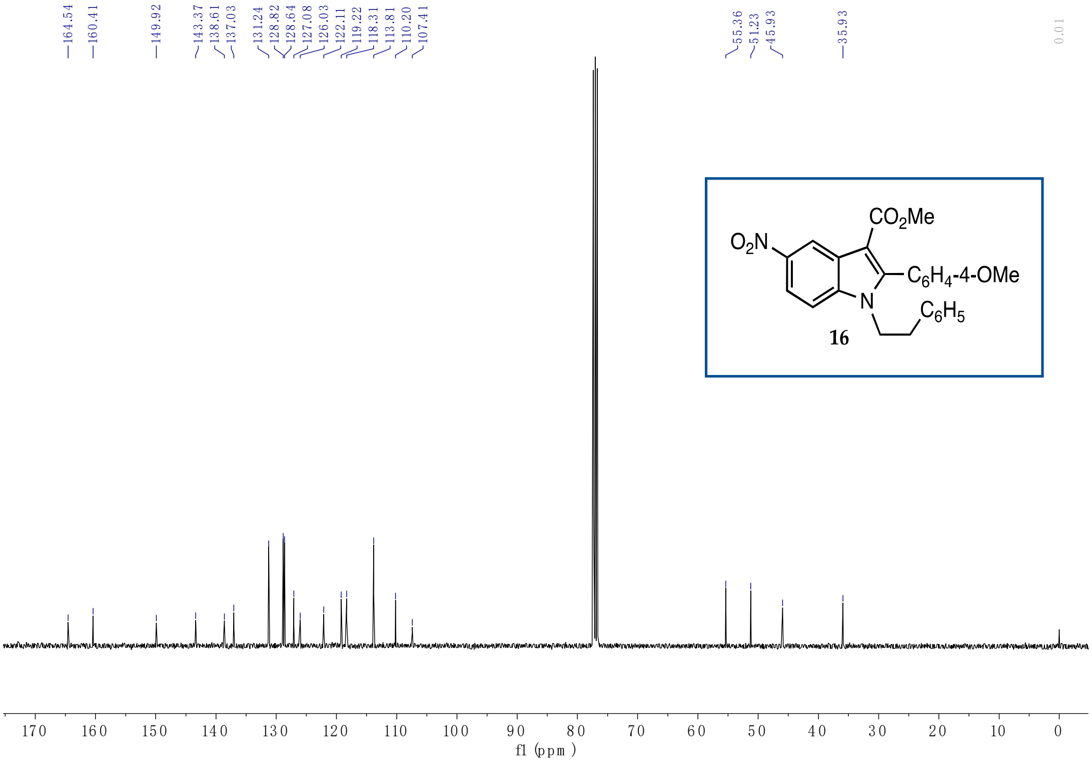

^{1}H and ^{13}C NMR for Methyl 1-Benzyl-5-cyano-2-phenyl-1*H*-indole-3-carboxylate (**17**)

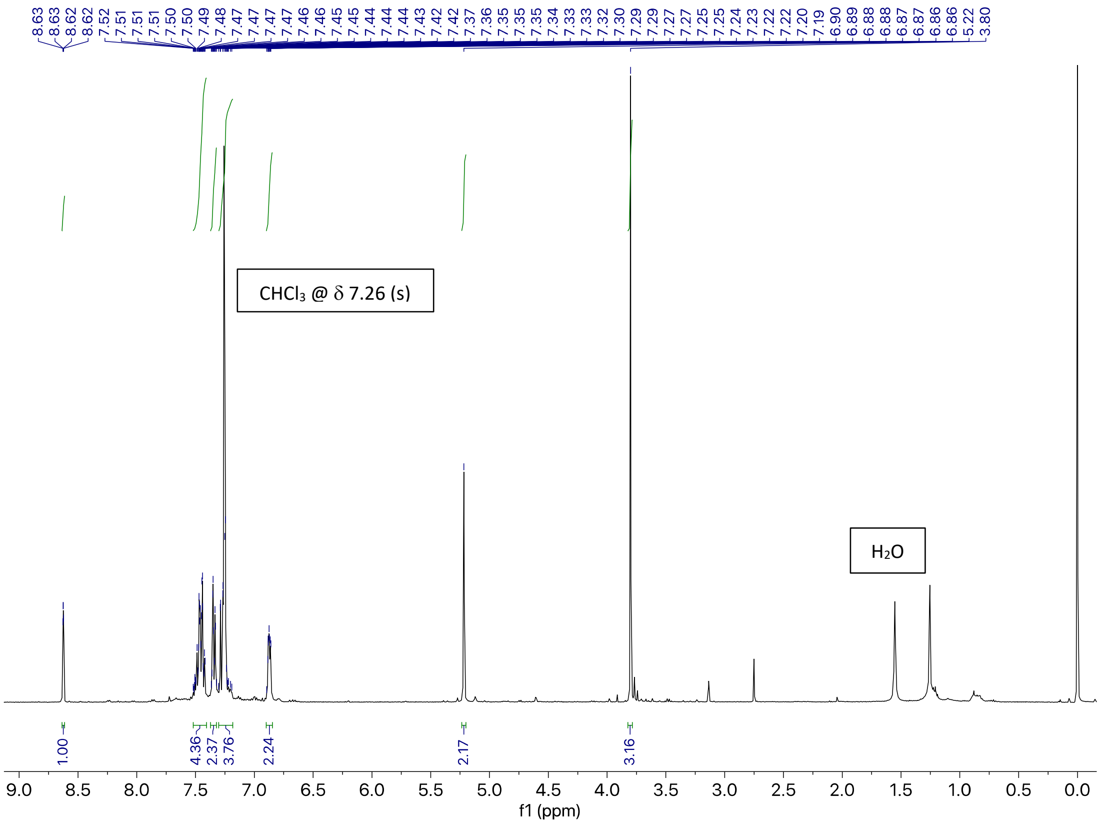

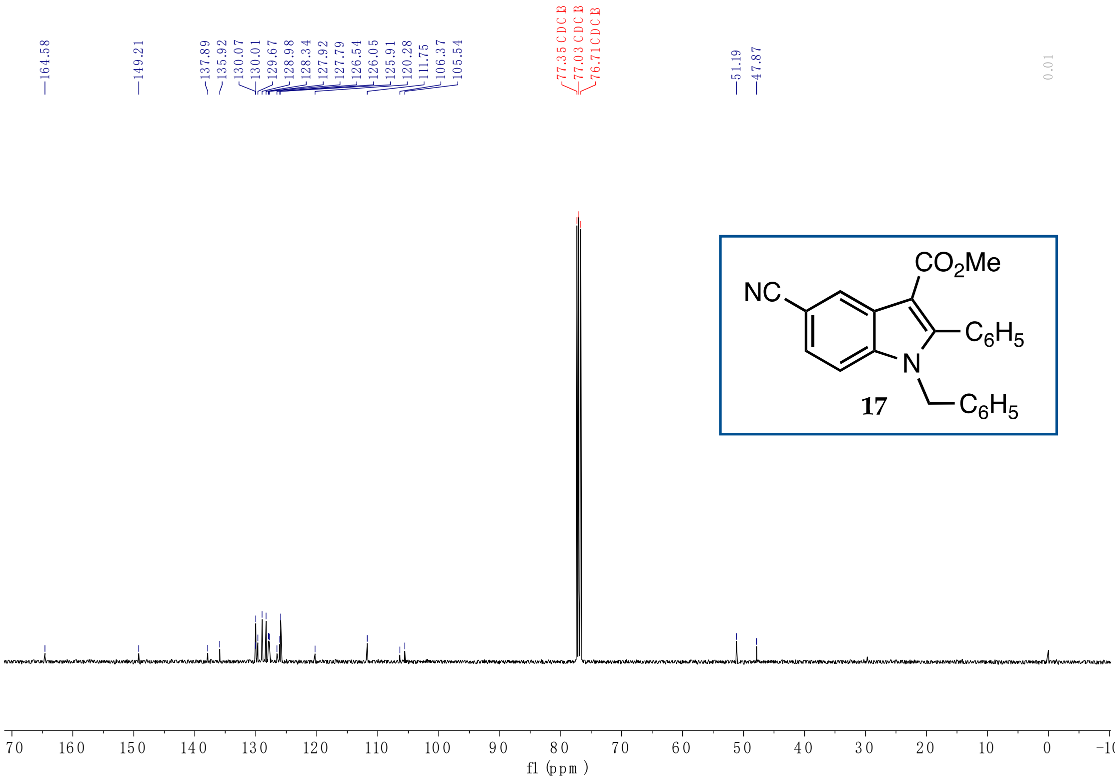

^{1}H and ^{13}C NMR for Methyl 5-Cyano-1-phenethyl-2-phenyl-1*H*-indole-3-carboxylate (**18**)

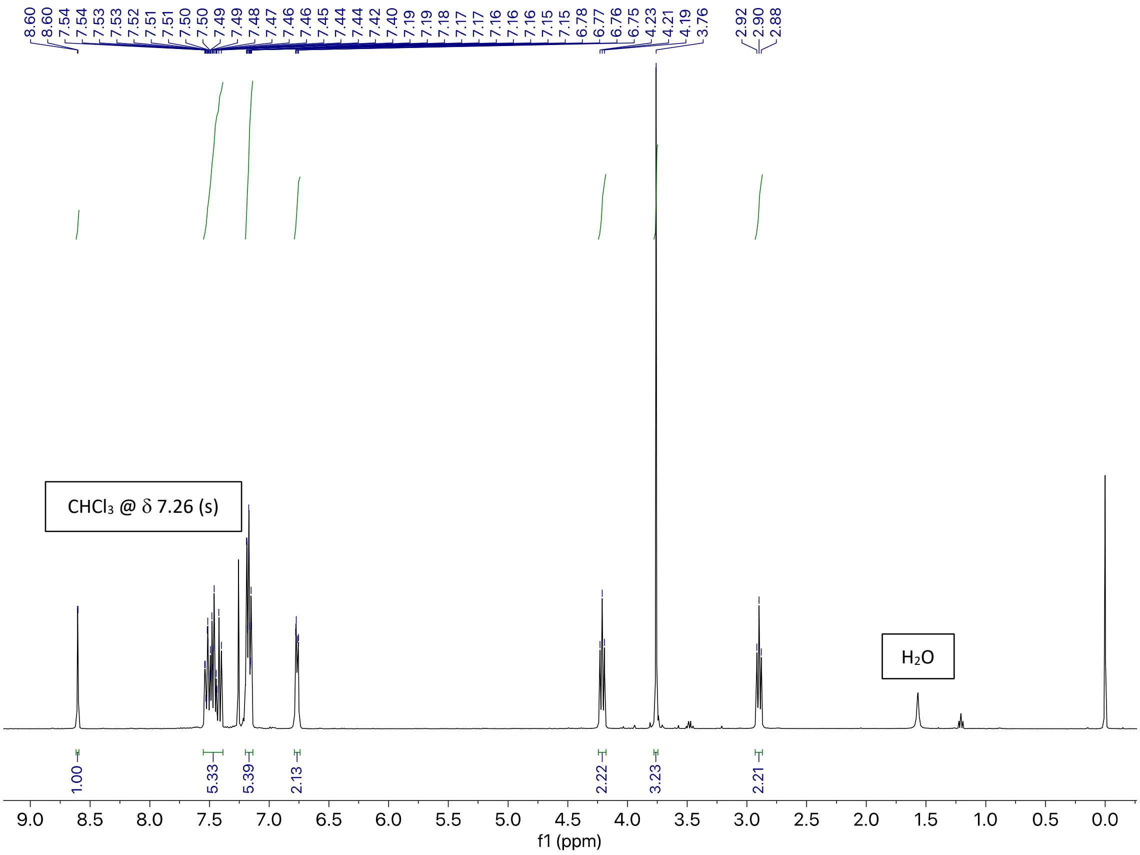
8.60 8.60 7.54 7.54 7.53 7.52 7.51 7.51 7.50 7.49 7.49 7.48 7.47 7.46 7.46 7.45 7.44 7.44 7.42 7.40 7.19 7.19 7.18 7.17 7.17 7.16 7.16 7.15 6.78 6.77 6.76 6.75 4.23 4.21 4.19 3.76 2.92 2.90 2.88
CHCl3 @ δ 7.26 (s)
H2O
1.00 5.33 5.39 2.13 2.22 3.23 2.21
9.0 8.5 8.0 7.5 7.0 6.5 6.0 5.5 5.0 4.5 4.0 3.5 3.0 2.5 2.0 1.5 1.0 0.5 0.0
f1 (ppm)

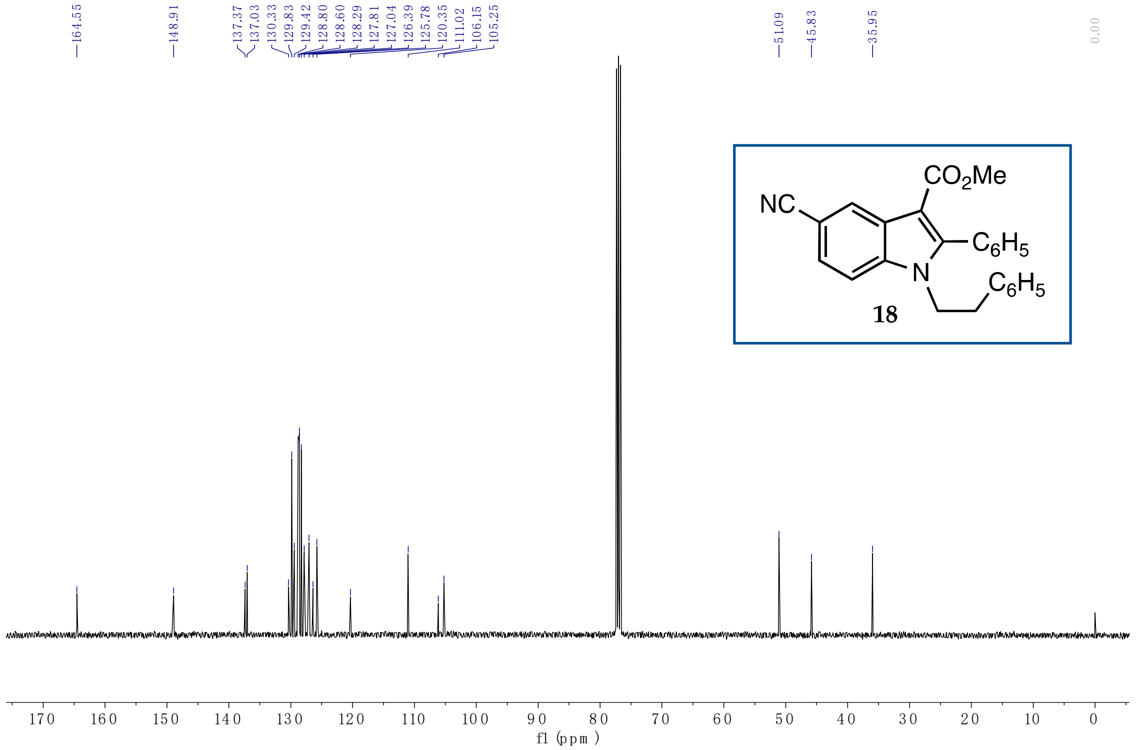
164.55 148.91 137.37 137.03 130.33 129.83 129.42 128.80 128.60 128.29 127.81 127.04 126.39 125.78 120.35 111.02 106.15 105.25 51.09 45.83 35.95 0.00
CO2Me
NC
C6H5
N
C6H5
18
170 160 150 140 130 120 110 100 90 80 70 60 50 40 30 20 10 0
f1 (ppm)

^{1}H and ^{13}C NMR for Methyl 5-Cyano-2-(4-methylphenyl)-1-phenethyl-1*H*-indole-3-carboxylate (**19**)

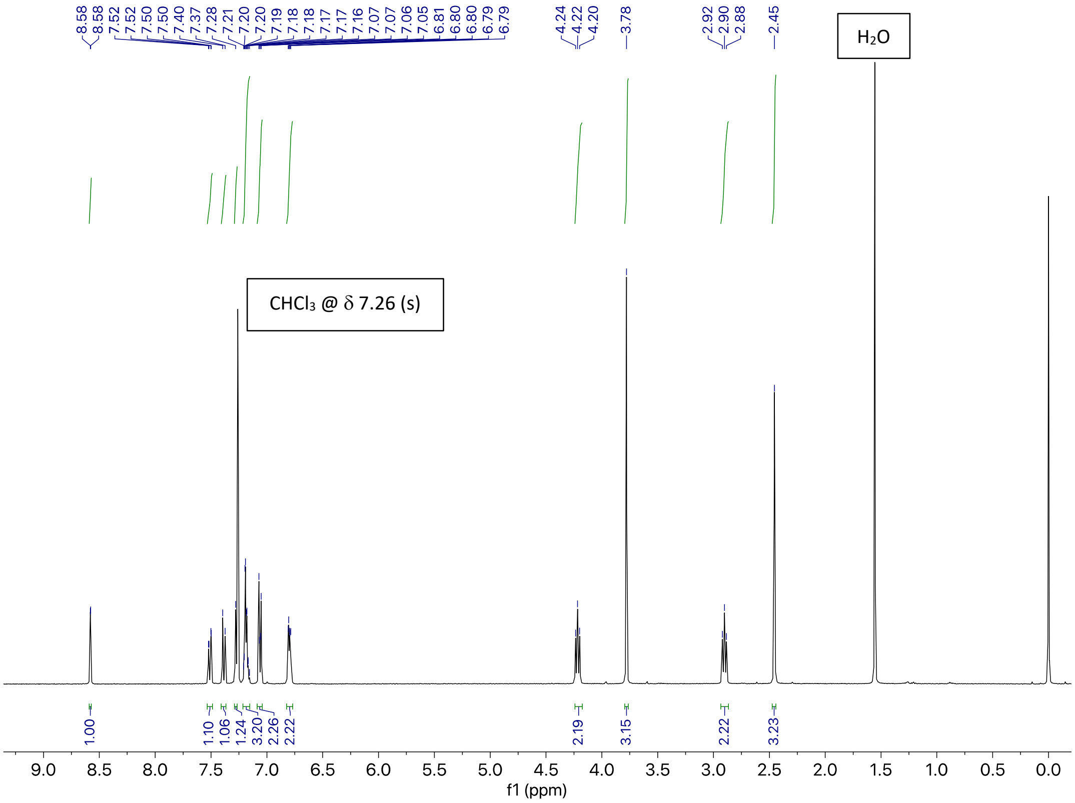

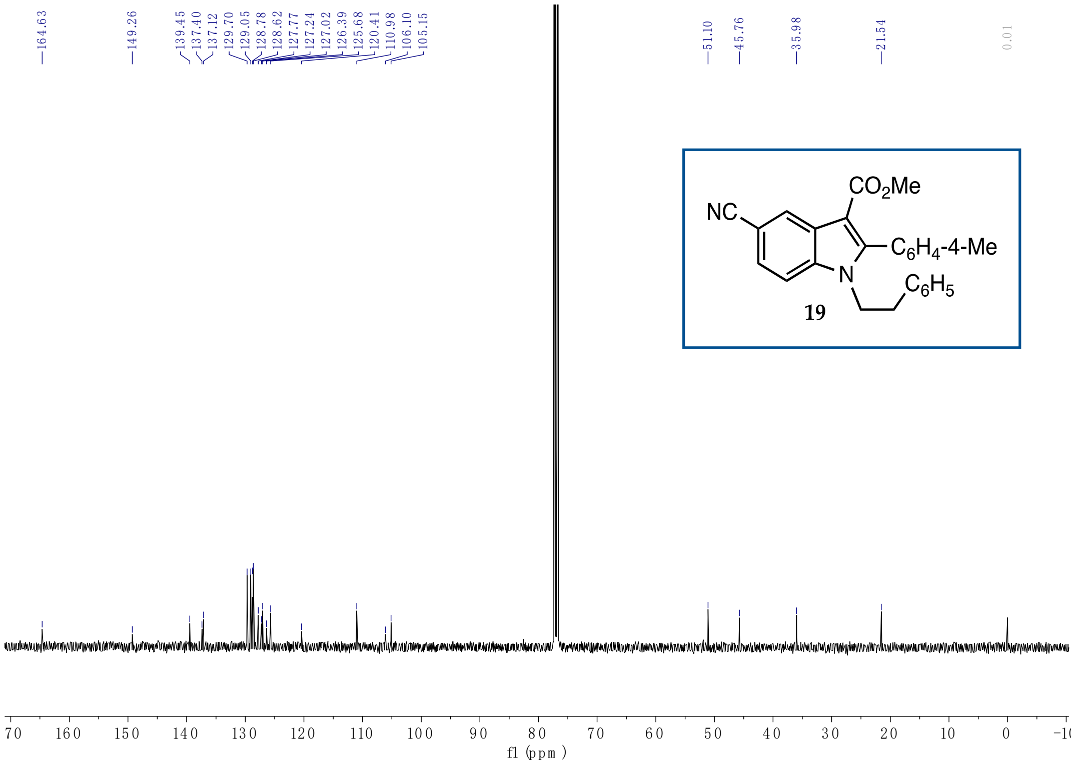

^{1}H and ^{13}C NMR for Methyl 5-Cyano-2-(4-fluorophenyl)-1-phenethyl-1*H*-indole-3-carboxylate (**20**)

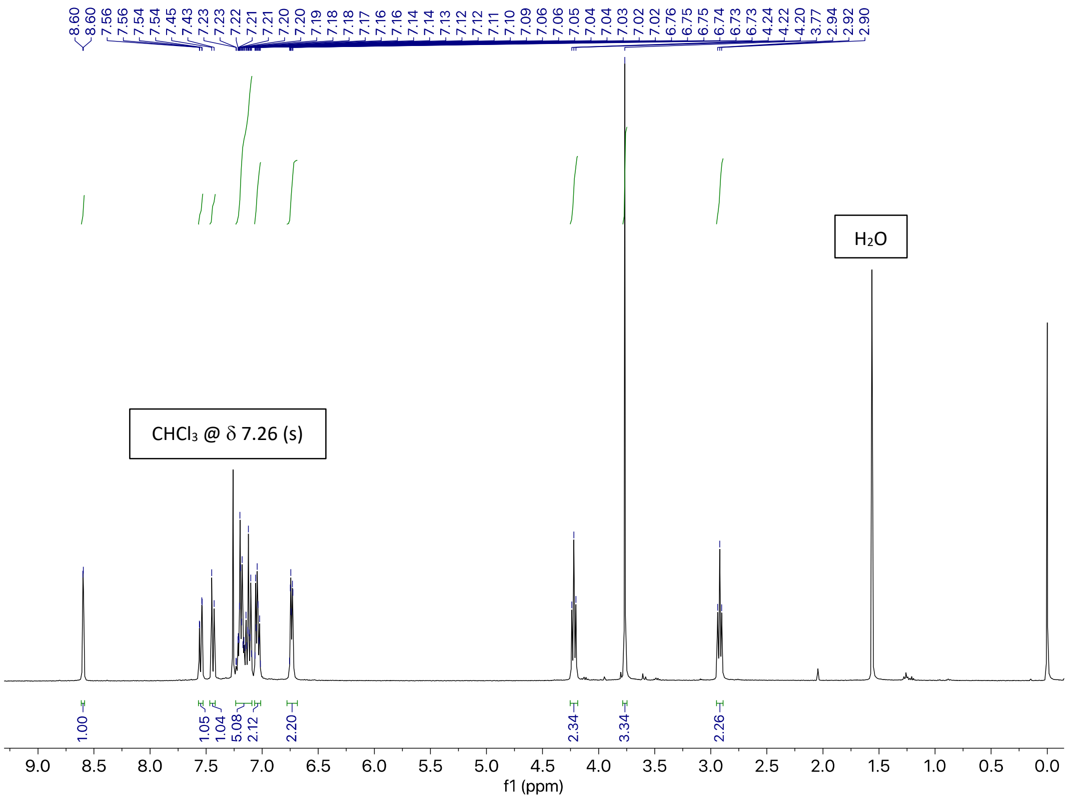

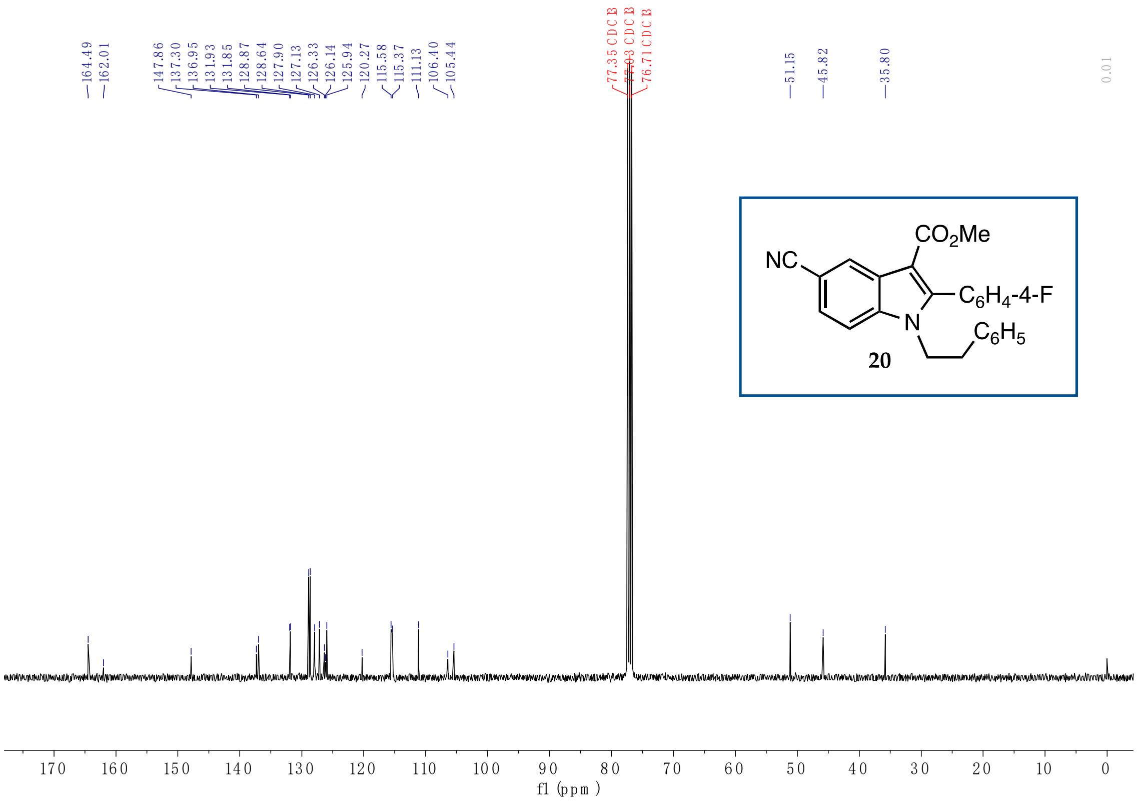

^{19}F NMR for Methyl 5-Cyano-2-(4-fluorophenyl)-1-phenethyl-1*H*-indole-3-carboxylate (**20**)

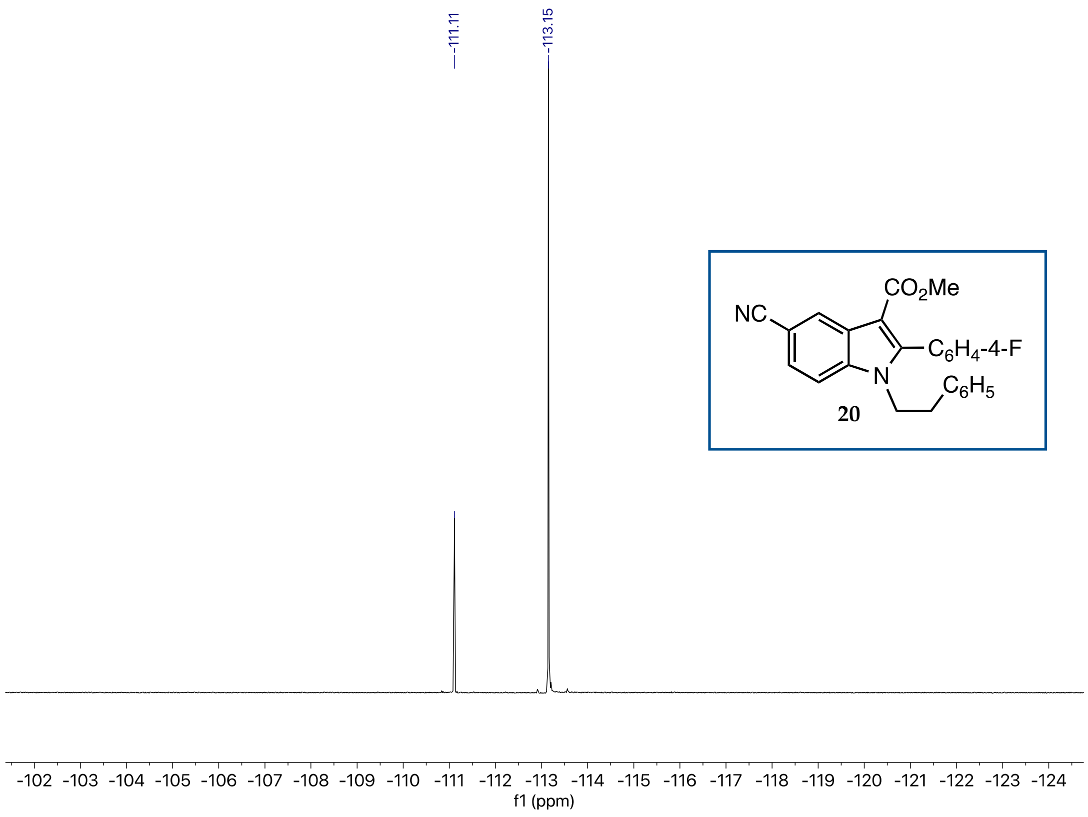

^{1}H and ^{13}C NMR for Methyl 2-(4-Chlorophenyl)-5-cyano-1-phenethyl-1*H*-indole-3-carboxylate (**21**)

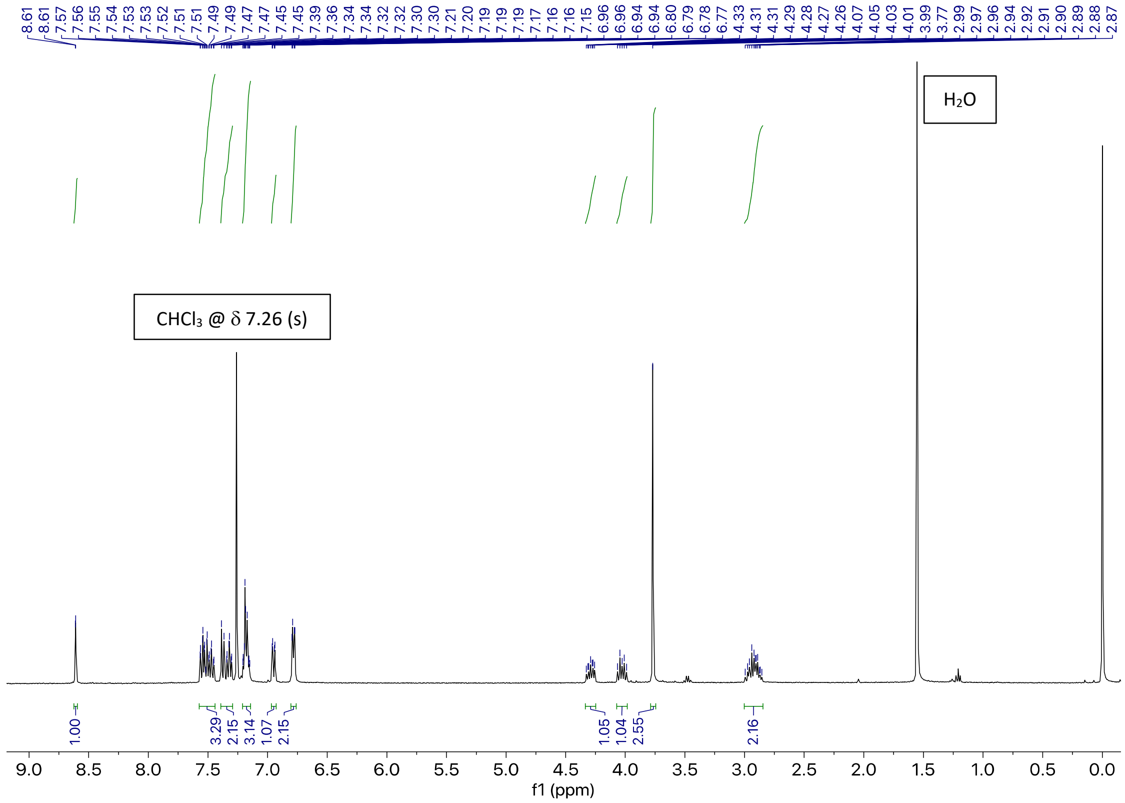

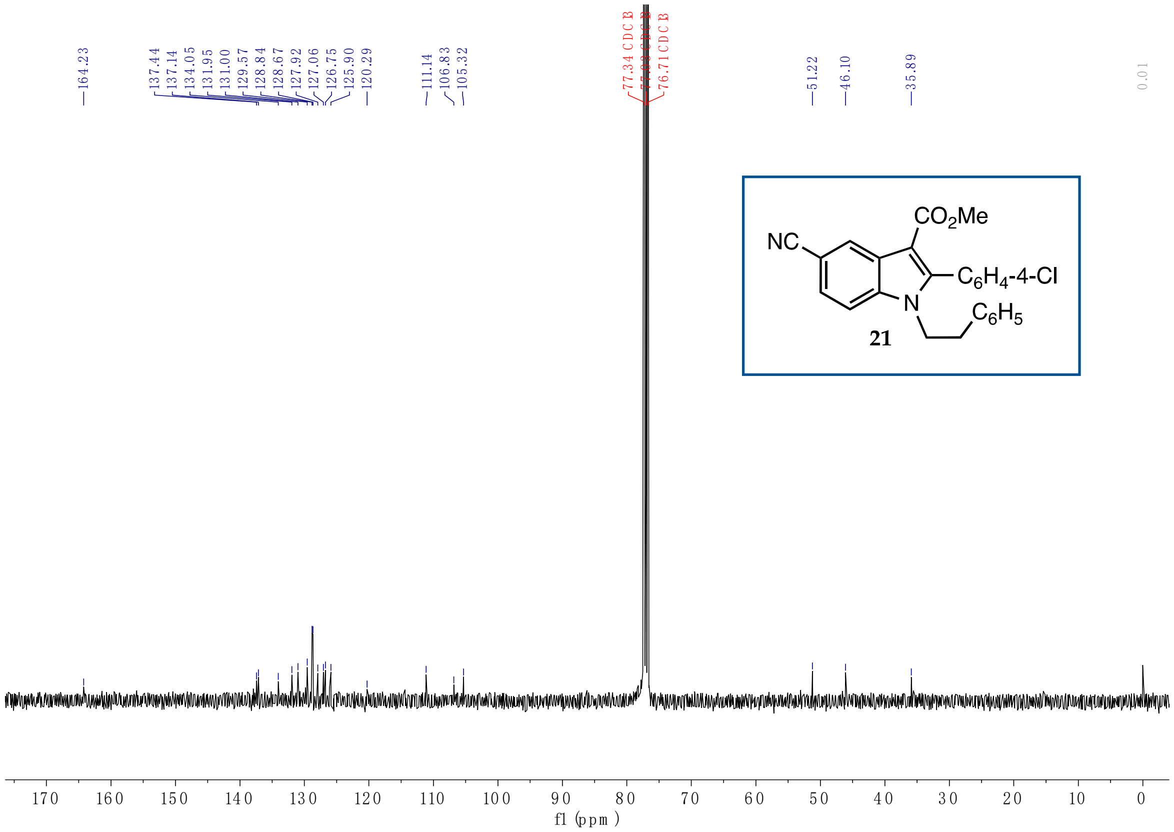

^{1}H and ^{13}C NMR for Methyl 5-Cyano-2-(4-methoxyphenyl)-1-phenethyl-1*H*-indole-3-carboxylate (**22**)

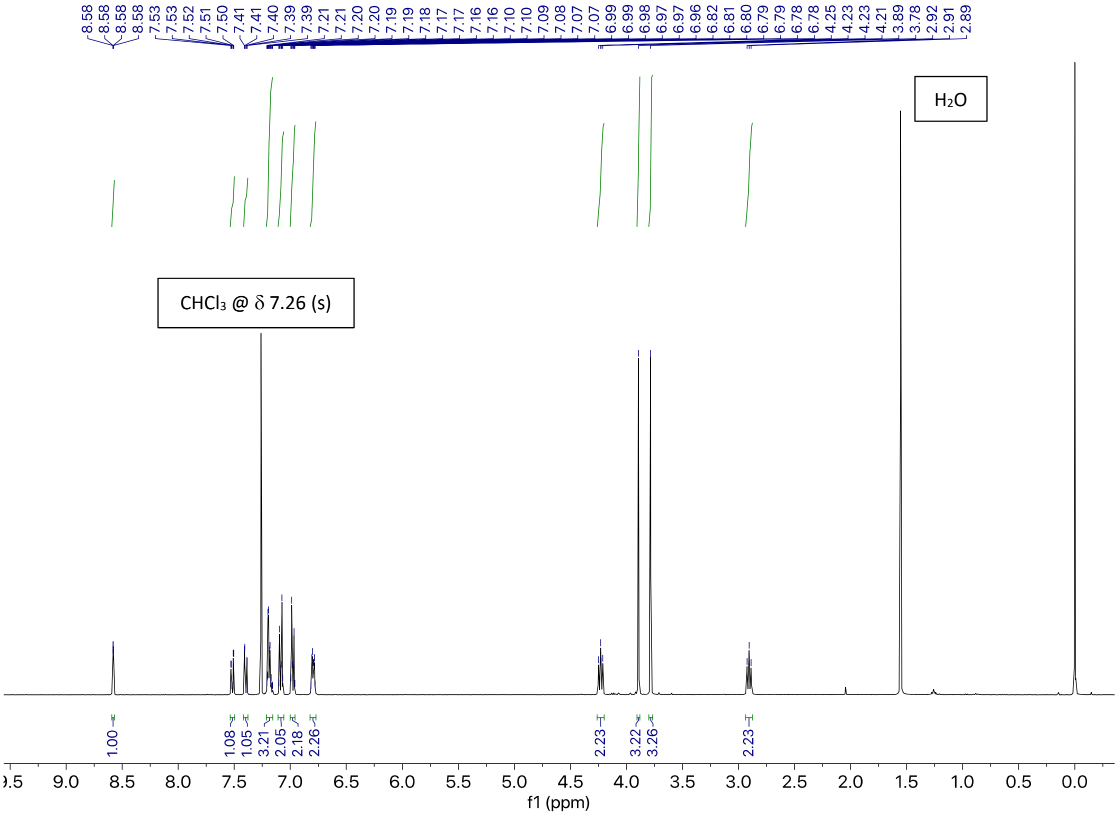
8.58 8.58 8.58 8.58 7.53 7.53 7.52 7.51 7.50 7.41 7.41 7.40 7.39 7.39 7.21 7.21 7.20 7.20 7.19 7.19 7.18 7.17 7.17 7.16 7.16 7.10 7.09 7.08 7.07 7.07 6.99 6.99 6.98 6.97 6.97 6.96 6.82 6.81 6.80 6.79 6.79 6.78 6.78 4.25 4.23 4.23 4.21 3.89 3.78 2.92 2.91 2.89
H2O
CHCl3 @ δ 7.26 (s)
1.00 1.08 1.05 3.21 2.05 2.18 2.26 2.23 3.22 3.26 2.23
f1 (ppm)

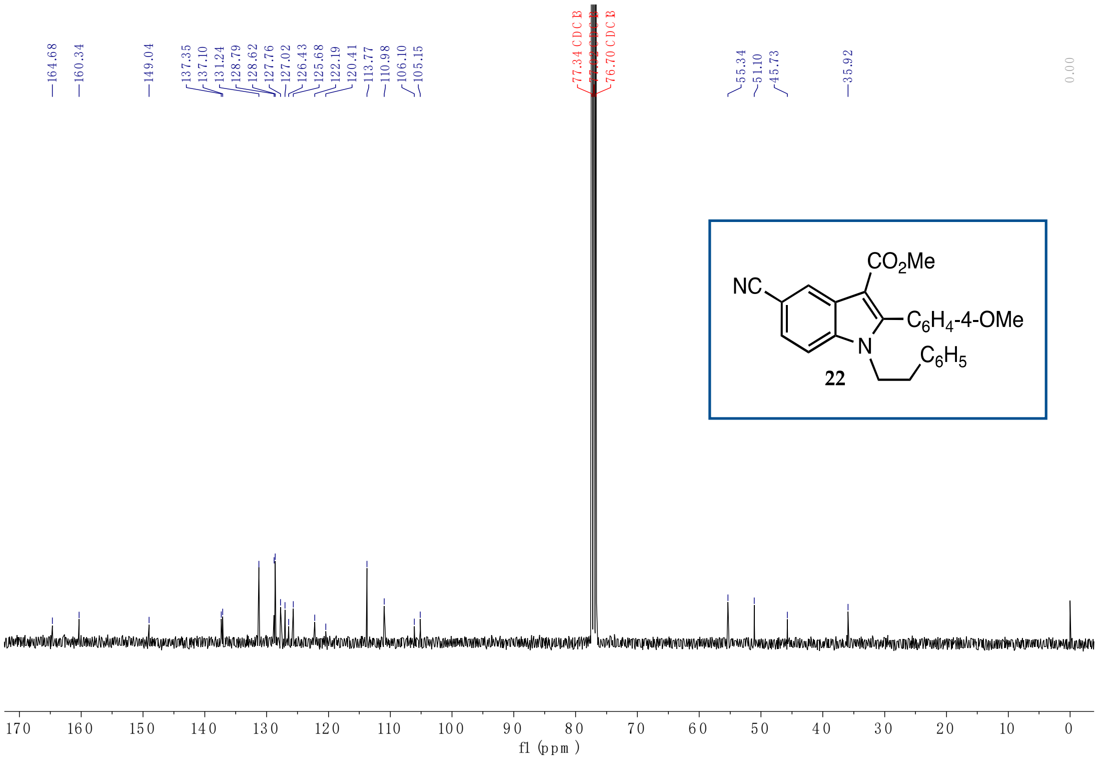
164.68 160.34 149.04 137.35 137.10 131.24 128.79 128.62 127.76 127.02 126.43 125.68 122.19 120.41 113.77 110.98 106.10 105.15
77.34 CDCl3
76.70 CDCl3
55.34 51.10 45.73 35.92 0.00
f1 (ppm)
CO2Me
NC
C6H4-4-OMe
N
C6H5
22
